# The performance of 11 fingertip pulse oximeters during hypoxemia in healthy human participants with varied, quantified skin pigment

**DOI:** 10.1016/j.ebiom.2024.105051

**Published:** 2024-03-08

**Authors:** Gregory Leeb, Isabella Auchus, Tyler Law, Philip Bickler, John Feiner, Shamsudini Hashi, Ellis Monk, Elizabeth Igaga, Michael Bernstein, Yu Celine Chou, Caroline Hughes, Deleree Schornack, Jenna Lester, Kelvin Moore, Olubunmi Okunlola, Jana Fernandez, Leonid Shmuylovich, Michael Lipnick

**Affiliations:** a Department of Anesthesia, University of California, San Francisco, USA; b Department of Sociology, Harvard University, USA; c Department of Anesthesia, College of Health Sciences, Makerere University, Uganda; d Physio Monitor, LLC, San Ramon, CA, USA; e Department of Dermatology, University of California, San Francisco, USA; f University of California, San Francisco School of Medicine, USA; g Department of Anesthesia, New York University Langone Hospital Brooklyn, USA; h Department of Dermatology, Washington University in St. Louis, USA; i University of California, San Francisco Institute for Global Health Sciences, USA

**Keywords:** Pulse oximeter, Skin pigmentation, Medical devices, Low-cost oximeter

## Abstract

**Background:**

Fingertip pulse oximeters are widely available, inexpensive, and commonly used to make clinical decisions in many settings. Device performance is largely unregulated and poorly characterised, especially in people with dark skin pigmentation.

**Methods:**

Eleven popular fingertip pulse oximeters were evaluated using the US Food and Drug Administration (FDA) Guidance (2013) and International Organization for Standardization Standards (ISO, 2017) in 34 healthy humans with diverse skin pigmentation utilising a controlled desaturation study with arterial oxygen saturation (SaO 2) plateaus between 70% and 100%. Skin pigmentation was assessed subjectively using a perceived Fitzpatrick Scale (pFP) and objectively using the individual typology angle (ITA) via spectrophotometry at nine anatomical sites.

**Findings:**

Five of 11 devices had a root mean square error (ARMS) > 3%, falling outside the acceptable FDA performance range. Nine devices demonstrated worse performance in participants in the darkest skin pigmentation category compared with those in the lightest category. A commonly used subjective skin colour scale frequently miscategorised participants as being darkly pigmented when compared to objective quantification of skin pigment by ITA.

**Interpretation:**

Fingertip pulse oximeters have variable performance, frequently not meeting regulatory requirements for clinical use, and occasionally contradicting claims made by manufacturers. Most devices showed a trend toward worse performance in participants with darker skin pigment. Regulatory standards do not adequately account for the impact of skin pigmentation on device performance. We recommend that the pFP and other non-standardised subjective skin colour scales should no longer be used for defining diversity of skin pigmentation. Reliable methods for characterising skin pigmentation to improve diversity and equitable performance of pulse oximeters are needed.

**Funding:**

This study was conducted as part of the Open Oximetry Project funded by the 10.13039/100000936Gordon and Betty Moore Foundation, Patrick J McGovern Foundation, and 10.13039/100000867Robert Wood Johnson Foundation. The UCSF Hypoxia Research Laboratory receives funding from multiple industry sponsors to test the sponsors' devices for the purposes of product development and regulatory performance testing. Data in this paper do not include sponsor's study devices. All data were collected from devices procured by the Hypoxia Research Laboratory for the purposes of independent research. No company provided any direct funding for this study, participated in study design or analysis, or was involved in analysing data or writing the manuscript. None of the authors own stock or equity interests in any pulse oximeter companies. Dr Ellis Monk's time utilised for data analysis, reviewing and editing was funded by grant number: DP2MH132941.

**Research in context**

**Evidence before this study**

Several studies have shown worse device performance in people with subjectively darker skin, using either self-reported race or a subjective scale to determine skin pigmentation of individuals, however, none have objectively measured skin pigmentation. Available data suggest poor root mean square error (ARMS) for many of these devices. Few prior studies have investigated the performance of inexpensive pulse oximeters with regard to the impact of skin pigment on device performance.

**Added value of this study**

This study utilises objective skin pigmentation measurement at various anatomical sites used for pulse oximetry. It compares them to the measurement with subjective scales, as well as testing more oximeters than prior studies. We found that a) many oximeters had variable performance, at times inconsistent with regulatory claims, b) current regulatory guidance does not adequately ensure diversity of skin pigmentation for device testing, and c) the optimal site for the objective measurement of skin pigment has not been defined and requires further investigation to ensure that pulse oximetry is valid for people with a diversity of skin tones.

**Implications of all the available evidence**

The findings of our study highlight the presence of variable pulse oximeter performance in the setting of nonspecific, non-harmonised regulatory requirements for inclusion of diverse individuals in device testing. Subjective, non-standardised skin colour scales should no longer be used for defining diversity of skin pigmentation in pulse oximeter validation studies. Attention should be given to creating standardised objective skin colour measurements used for regulatory testing in order to improve equity in device performance.

## Introduction

Pulse oximetry is globally recognised as an essential tool across a variety of clinical settings. Recently, there has been a dramatic increase in low-cost, fingertip pulse oximeter (POX) devices on the market.^1^^,^^2^ Despite this increase, an estimated 15% of operating rooms, 42% of post-anaesthesia care unit beds, and up to 64% of healthcare facilities in low-income and middle-income countries (LMICs) lack access to POXs.^3^^,^^4^ Inexpensive POXs are regularly utilised in hospital and home settings, particularly in LMICs, often without adequate device performance information. LMICs are disproportionately affected due to an unprecedented volume of COVID-19 related donations and the increased likelihood of cost impacting POX procurement decisions. There are significant safety concerns with these devices which are frequently and inappropriately used for clinical decision making.^5^, ^6^, ^7^, ^8^, ^9^ Many of these devices do not adhere to regulatory standards, at times making inaccurate claims about performance. Few have been thoroughly and independently investigated, especially regarding the impact of skin pigment on device performance.^5^ Collectively, these issues have made pulse oximetry a specific focus of the Lancet Global Health Commission on Medical Oxygen Security.^10^

Since the late 1980's, studies have demonstrated that POX performance is impacted by dark skin pigmentation.^6^^,^^7^ Studies performed on healthy volunteers have demonstrated that POXs in darkly pigmented, hypoxemic individuals exhibited a greater positive bias in peripheral oxygen saturation (SpO 2), greater than the recorded functional arterial oxygen saturation (SaO 2) compared to lightly pigmented individuals.^11^, ^12^, ^13^ In recent years and during the COVID-19 pandemic, multiple studies from the clinical setting demonstrated positive bias for SpO2 as well as associations with delayed, withheld treatments and increased readmission rates for patients with darker skin pigment or who self-identify as black.^14^, ^15^, ^16^, ^17^, ^18^

The Food and Drug Administration (FDA) and International Organization for Standardization (ISO) currently require POXs to be tested on at least ten healthy humans, and the FDA requires at least two or 15% of these individuals to be ‘darkly pigmented’. However, the small proportion of participants with dark pigment as well as the lack of definition for the term ‘darkly pigmented’ have been flagged as problematic and possible contributing factors to pulse oximetry bias.^19^ Studies assessing the effect of skin pigment on POX accuracy, including those from our laboratory^20^ and recent clinical studies,^14^^,^^15^ have failed to objectively measure skin pigmentation and have instead relied on either non-standardised subjective colour scales or self-reported ethnicity or race.^13^ The Fitzpatrick Scale in particular, which is a question-based and non-visual assessment of the propensity of sun-exposed skin to burn and tan, is one of the most commonly used subjective measures of skin pigmentation for POX studies.^21^ Though not intended to be a visual analogue scale, it is common practice to estimate a perceived Fitzpatrick skin phototype (pFP) by subjectively matching an individual's perceived facial skin colour with a non-standardised visual analogue scale, ranging from I-VI. Significant concerns regarding the pFP have been previously raised, including the subjective and non-standardised nature of the assessment and inadequate representation of darker skin types.^22^ Furthermore, in the context of POX validation studies, the pFP is usually estimated by visual assessment of the forehead or cheek and may not accurately approximate the optical properties at the site of POX measurement (e.g. fingertip or ear).

With low-cost POXs flooding the market via consumer procurement and donations to LMICs, and renewed concerns about performance problems in patients with darkly pigmented skin, we aimed to assess the performance of 11 popular fingertip oximeters using current regulatory requirements for device performance. We also sought to compare subjective skin pigmentation assessment with objective skin pigmentation assessment by spectrophotometry, and to determine if using pFP to ensure diverse skin pigmentation in regulatory testing is adequate.

## Methods

We tested 11 fingertip POXs and a laboratory tabletop POX (used for participant monitoring), referred to as the clinical reference device, in 34 healthy participants who volunteered for pulse oximetry controlled desaturation studies at the UCSF Hypoxia Research Laboratory from 2022 to 2023. Participants were adults, non-smokers and without lung disease, obesity, or cardiovascular comorbidities. Gender was self-reported by study participants. Demographic data on the study population are presented in Table 1.

**Table 1:** Demographic information.

n	34
Age (years) Median (IQR Q1, Q3)	26.5 (23, 30)
Gender	
Female	17 (50%)
Male	17 (50%)
Ethnicity	
Asian	9 (27%)
Black	7 (21%)
Caucasian	8 (24%)
Hispanic	5 (15%)
Multiethnic	5 (15%)
Fitzpatrick scale	
I	1 (3%)
II	2 (6%)
III	12 (35%)
IV	10 (29%)
V	5 (15%)
VI	4 (12%)
ITA° Median (IQR Q1, Q3); Range	
Forehead	19.5 (−4.3, 30); (−65.7, 44.7)
Dorsal DP	23.4 (−4.2, 30.6); (−67, 46.8)
Inner upper arm	32.2 (14, 40); (−54.7, 62.3)
BMI (kg/m2)	
<20	6 (18%)
20–25	20 (59%)
26–30	7 (21%)

Pulse oximeters tested are listed in Table 2, and further details surrounding these devices can be found at the OpenOximetry.org website.^23^ Nine of the 11 devices cost less than $60 USD; the Masimo MightySat and Nonin Onyx Vantage 9590 cost approximately $199 USD. Study devices were selected based on popularity in online marketplaces and discussions about device availability with providers from diverse practice settings. Four devices had FDA 510(k) premarket notification clearance (Contec–K082641, Biolight–K151287, Masimo–K181956, Nonin–K112843).^24^ These four devices also report to have met ISO standard 80601-2-61.

**Table 2:** Device data by SaO 2 plateau.

	70–100%	Lightest Third			Medium Third			Darkest Third		
		70–80%	80–90%	90–100%	70–80%	80–90%	90–100%	70–80%	80–90%	90–100%
**Number of SaO 2 measurements**										
Nellcor (Reference)	1084	112	124	78	140	153	95	134	153	95
Nonin Onyx Vantage 9590	332	24	40	42	39	33	47	27	40	40
Masimo Mightysat	329	36	42	16	36	56	18	41	61	23
Walgreens MD300CN350R	309	30	38	14	37	57	15	42	59	17
Zacurate CMS 500DL	223	16	28	13	20	30	30	30	32	24
Walgreens OxyWatch C20	289	34	27	19	33	43	24	42	39	28
Choice MMed MD300CN340	309	36	35	16	37	52	18	41	51	23
Zacurate 500C	327	32	42	16	35	57	18	43	61	23
Bodymed BDMOXMTRBLK	224	16	26	9	20	40	21	30	42	20
Roscoe POX-ROS	326	28	30	17	40	47	33	42	53	36
CONTEC CMS50M	287	31	38	16	26	49	18	35	51	23
Biolight M70	321	49	24	21	64	28	33	49	24	29
**Bias (95% CI, %)**										
Nellcor (Reference)	1.1 (1.0, 1.2)	0.4 (0.0, 0.7)	1.1 (0.8, 1.4)	3.0 (2.4, 3.5)	1.0 (0.7, 1.2)	1.6 (1.2, 2.0)	1.7 (1.4, 1.9)	0.4 (0.2, 0.7)	0.7 (0.5, 0.9)	0.5 (0.2, 0.8)
Nonin Onyx Vantage 9590	0.6 (0.4, 0.8)	0.5 (−0.2, 1.2)	0.9 (0.4, 1.3)	2.0 (1.7, 2.4)	0.0 (−0.3, 0.4)	1.1 (0.5, 1.9)	0.8 (0.5, 1.2)	0.1 (−0.5, 0.6)	0.2 (−0.7, 1.0)	−0.2 (−1.3, 0.2)
Masimo Mightysat	0.7 (0.5, 0.9)	0.3 (−0.3, 0.8)	0.9 (0.1, 2.0)	4.0 (3.1, 4.8)	0.6 (0.2, 1.1)	0.4 (0.1, 0.8)	2.4 (1.9, 3.1)	−0.1 (−0.8, 0.3)	0.0 (−0.2, 0.2)	1.6 (1.1, 2.3)
Walgreens MD300CN350R	0.8 (0.6, 1.1)	0.4 (−0.4, 1.1)	0.4 (−0.6, 1.3)	4.3 (2.8, 5.7)	0.9 (0.4, 1.4)	1.1 (0.8, 1.5)	3.2 (2.2, 4.2)	−0.8 (−1.2, −0.4)	0.7 (0.4, 1.1)	0.6 (−2.3, 1.7)
Zacurate CMS 500DL	1.6 (1.3, 1.8)	1.8 (0.7, 2.6)	1.1 (−0.0, 2.0)	4.2 (3.4, 4.9)	2.0 (1.4, 2.5)	1.9 (0.9, 2.5)	2.4 (1.9, 2.8)	−0.1 (−0.6, 0.4)	1.7 (1.3, 2.2)	0.6 (0.1, 1.3)
Walgreens OxyWatch C20	1.3 (1.0, 1.6)	0.8 (0.1, 1.5)	0.8 (−0.1, 1.6)	4.0 (2.0, 6.8)	1.4 (0.8, 1.8)	1.6 (1.1, 2.0)	2.7 (1.9, 3.7)	−0.0 (−0.6, 0.4)	0.9 (0.5, 1.4)	1.0 (0.7, 1.3)
Choice MMed MD300CN340	1.6 (1.4, 1.9)	2.4 (1.4, 3.5)	2.2 (1.7, 2.8)	2.5 (1.2, 3.6)	2.0 (1.5, 2.6)	1.7 (1.4, 2.1)	2.1 (−0.1, 3.1)	1.0 (0.5, 1.5)	0.6 (0.3, 0.8)	1.2 (−0.3, 2.2)
Zacurate 500C	−0.0 (−0.4, 0.3)	0.1 (−1.4, 1.3)	−1.1 (−2.0, −0.2)	−3.6 (−8.5, 1.1)	1.8 (1.3, 2.2)	0.3 (−0.1, 0.7)	1.0 (−1.1, 2.9)	0.3 (−0.1, 0.7)	−0.5 (−0.8, −0.2)	0.7 (−0.2, 1.7)
Bodymed BDMOXMTRBLK	2.0 (1.6, 2.4)	2.5 (0.1, 4.1)	4.2 (2.7, 6.0)	5.1 (3.8, 6.1)	1.9 (0.9, 2.8)	1.7 (0.8, 2.5)	3.1 (2.6, 3.6)	−0.1 (−0.7, 0.4)	1.5 (0.9, 2.1)	0.9 (0.5, 1.3)
Roscoe POX-ROS	−0.9 (−1.4, −0.5)	−0.8 (−1.4, −0.2)	−0.8 (−1.5, 0.1)	2.7 (−0.1, 6.7)	−0.1 (−0.7, 0.6)	−1.1 (−1.9, −0.4)	−3.6 (−6.8, −0.5)	−1.1 (−1.5, −0.6)	0.4 (−0.1, 0.9)	−3.1 (−4.1, −2.3)
CONTEC CMS50M	−2.0 (−2.5, −1.6)	−4.0 (−5.7, −2.5)	−2.6 (−3.7, −0.6)	−3.4 (−6.9, −0.4)	−2.0 (−3.5, −0.6)	−2.8 (−3.4, −2.3)	−0.1 (−1.8, 1.3)	−0.9 (−1.9, 0.1)	−1.3 (−1.9, −0.7)	−0.4 (−1.8, 0.6)
Biolight M70	0.2 (−0.3, 0.6)	−2.0 (−3.6, 0.0)	−1.1 (−3.3, 1.8)	1.5 (−0.5, 3.7)	1.0 (0.2, 1.7)	0.2 (−1.3, 2.5)	−1.0 (−2.6, 0.0)	1.8 (0.7, 2.5)	−0.6 (−2.2, 0.6)	1.0 (0.7, 1.3)
**|Bias| (95% CI, %)**										
Nellcor (Reference)	1.6 (1.5, 1.7)	1.6 (1.4, 1.8)	1.8 (1.6, 2.0)	3.3 (2.9, 3.8)	1.3 (1.1, 1.5)	2.0 (1.7, 2.4)	1.7 (1.5, 2.0)	1.2 (1.0, 1.3)	1.1 (1.0, 1.3)	1.1 (0.9, 1.3)
Nonin Onyx Vantage 9590	1.4 (1.2, 1.5)	1.7 (1.4, 2.0)	1.4 (1.1, 1.7)	2.0 (1.7, 2.4)	0.9 (0.7, 1.2)	1.5 (1.1, 2.2)	1.1 (0.8, 1.4)	1.0 (0.7, 1.5)	1.9 (1.3, 2.7)	0.9 (0.6, 2.0)
Masimo Mightysat	1.4 (1.3, 1.6)	1.4 (1.1, 1.7)	2.0 (1.4, 3.0)	4.0 (3.1, 4.8)	1.1 (0.8, 1.5)	1.0 (0.7, 1.3)	2.4 (1.9, 3.0)	1.4 (1.1, 1.9)	0.7 (0.6, 0.9)	1.7 (1.2, 2.4)
Walgreens MD300CN350R	1.7 (1.6, 1.9)	1.8 (1.4, 2.2)	2.4 (1.9, 3.0)	4.4 (2.9, 5.8)	1.4 (1.1, 1.8)	1.5 (1.2, 1.8)	3.2 (2.2, 4.2)	1.2 (1.0, 1.6)	0.9 (0.7, 1.3)	2.4 (1.5, 4.7)
Zacurate CMS 500DL	2.1 (1.9, 2.3)	2.3 (1.7, 3.0)	2.3 (1.7, 3.1)	4.2 (3.4, 4.9)	2.0 (1.5, 2.5)	2.4 (2.0, 2.9)	2.4 (2.0, 2.8)	1.1 (0.9, 1.4)	1.8 (1.4, 2.3)	1.2 (0.9, 1.7)
Walgreens OxyWatch C20	1.8 (1.6, 2.1)	1.9 (1.5, 2.3)	2.0 (1.5, 2.5)	4.0 (2.0, 6.7)	1.7 (1.4, 2.0)	1.7 (1.4, 2.1)	2.7 (1.9, 3.7)	1.2 (0.9, 1.6)	1.3 (1.0, 1.7)	1.2 (0.9, 1.4)
Choice MMed MD300CN340	2.1 (1.9, 2.3)	3.5 (2.8, 4.3)	2.3 (1.9, 2.9)	3.0 (2.1, 3.8)	2.1 (1.6, 2.7)	1.8 (1.5, 2.1)	3.5 (3.1, 4.2)	1.5 (1.1, 1.9)	0.8 (0.7, 1.0)	2.4 (1.7, 3.4)
Zacurate 500C	2.3 (2.0, 2.6)	3.0 (2.3, 4.1)	2.4 (1.8, 3.1)	9.8 (7.8, 12.4)	2.0 (1.7, 2.4)	1.5 (1.2, 1.7)	4.1 (3.3, 4.7)	1.1 (0.9, 1.4)	1.1 (0.9, 1.4)	2.0 (1.4, 2.7)
Bodymed BDMOXMTRBLK	2.6 (2.4, 3.0)	3.8 (2.4, 5.1)	4.8 (3.6, 6.4)	5.1 (3.9, 6.1)	2.4 (1.7, 3.1)	2.7 (2.2, 3.4)	3.1 (2.7, 3.6)	1.1 (0.8, 1.5)	2.0 (1.5, 2.5)	1.1 (0.8, 1.4)
Roscoe POX-ROS	2.5 (2.2, 3.0)	1.5 (1.2, 1.8)	1.7 (1.2, 2.4)	5.2 (3.1, 8.6)	1.8 (1.5, 2.2)	2.0 (1.5, 2.6)	7.2 (5.1, 9.7)	1.4 (1.1, 1.7)	1.3 (1.0, 1.8)	3.1 (2.3, 4.1)
CONTEC CMS50M	3.1 (2.8, 3.5)	5.1 (4.0, 6.3)	3.7 (2.7, 5.3)	5.5 (3.4, 8.3)	3.5 (2.6, 4.7)	2.8 (2.3, 3.4)	2.8 (2.0, 3.8)	2.5 (2.1, 3.3)	2.0 (1.6, 2.5)	2.0 (1.3, 3.0)
Biolight M70	3.2 (2.9, 3.6)	5.5 (4.6, 6.6)	4.5 (3.1, 6.5)	4.1 (2.9, 5.6)	2.6 (2.2, 3.2)	3.7 (2.8, 5.4)	2.0 (1.2, 3.5)	2.7 (2.3, 3.5)	2.5 (1.5, 3.7)	1.1 (0.9, 1.4)
**Precision (%)**										
Nellcor (Reference)	1.9	1.9	1.9	2.6	1.4	2.3	1.4	1.4	1.3	1.4
Nonin Onyx Vantage 9590	1.9	1.8	1.5	1.1	1.2	2.0	1.3	1.5	2.9	2.0
Masimo Mightysat	2.0	1.7	3.2	1.8	1.4	1.4	1.3	1.9	0.9	1.4
Walgreens MD300CN350R	2.3	2.1	3	2.9	1.5	1.5	2	1.4	1.3	3.8
Zacurate CMS 500DL	2.0	2.0	2.8	1.4	1.2	2.0	1.3	1.3	1.3	1.5
Walgreens OxyWatch C20	2.3	2.1	2.2	5.4	1.3	1.4	2.3	1.6	1.4	0.9
Choice MMed MD300CN340	2.1	3.4	1.6	2.5	1.8	1.3	3.1	1.7	0.8	2.9
Zacurate 500C	3.4	3.9	3.0	10.6	1.5	1.7	4.4	1.4	1.3	2.5
Bodymed BDMOXMTRBLK	2.9	4.1	4.4	1.8	2.2	2.9	1.1	1.4	2.1	0.9
Roscoe POX-ROS	4.2	1.5	2.2	7.3	2.1	2.6	9.3	1.4	1.9	2.8
CONTEC CMS50M	3.8	4.6	4.6	6.9	3.9	2.0	3.5	3.0	2.3	2.9
Biolight M70	4.5	6.4	6.2	5.1	3.1	5.0	3.7	2.9	3.7	0.8
**ARMS (95% CI, %)**										
Nellcor (Reference)	2.2 (2.0, 2.3)	1.9 (1.7, 2.1)	2.2 (2.0, 2.5)	3.9 (3.4, 4.4)	1.7 (1.5, 1.9)	2.8 (2.3, 3.6)	2.1 (1.9, 2.5)	1.5 (1.3, 1.7)	1.5 (1.3, 1.7)	1.5 (1.3, 1.8)
Nonin Onyx Vantage 9590	2.0 (1.8, 2.3)	1.8 (1.6, 2.3)	1.7 (1.4, 2.1)	2.3 (2.0, 2.7)	1.2 (1.0, 1.5)	2.2 (1.6, 3.0)	1.5 (1.2, 2.0)	1.4 (1.0, 2.0)	2.9 (2.1, 4.1)	2.0 (0.7, 4.5)
Masimo Mightysat	2.1 (1.9, 2.4)	1.7 (1.4, 2.1)	3.3 (2.3, 4.4)	4.3 (3.5, 5.1)	1.5 (1.2, 2.0)	1.4 (1.1, 1.9)	2.7 (2.1, 3.4)	1.8 (1.5, 2.7)	0.9 (0.7, 1.3)	2.1 (1.5, 3.3)
Walgreens MD300CN350R	2.4 (2.2, 2.8)	2.1 (1.8, 2.6)	3.0 (2.5, 3.6)	5.2 (3.9, 6.4)	1.7 (1.4, 2.1)	1.8 (1.5, 2.3)	3.7 (2.8, 4.6)	1.6 (1.3, 2.0)	1.4 (1.1, 1.9)	3.8 (2.1, 7.2)
Zacurate CMS 500DL	2.5 (2.3, 2.8)	2.7 (2.1, 3.6)	3.0 (2.3, 4.2)	4.4 (3.7, 5.1)	2.3 (1.9, 2.7)	2.8 (2.3, 3.4)	2.7 (2.4, 3.1)	1.3 (1.1, 1.6)	2.2 (1.8, 2.6)	1.6 (1.2, 2.1)
Walgreens OxyWatch C20	2.6 (2.2, 3.3)	2.2 (1.9, 2.7)	2.3 (1.9, 2.9)	6.6 (4.0, 9.3)	1.9 (1.6, 2.3)	2.1 (1.8, 2.5)	3.5 (2.6, 4.6)	1.6 (1.1, 2.4)	1.7 (1.4, 2.1)	1.4 (1.1, 1.7)
Choice MMed MD300CN340	2.7 (2.5, 2.9)	4.1 (3.3, 5.0)	2.7 (2.3, 3.3)	3.5 (2.7, 4.3)	2.7 (2.1, 3.4)	2.2 (1.8, 2.5)	3.7 (3.2, 4.5)	1.9 (1.6, 2.5)	1.0 (0.8, 1.1)	3.1 (2.3, 4.4)
Zacurate 500C	3.4 (2.9, 4.1)	3.9 (2.9, 5.3)	3.1 (2.5, 3.9)	10.8 (8.6, 13.2)	2.3 (1.9, 2.8)	1.7 (1.5, 2.0)	4.4 (3.7, 4.9)	1.4 (1.2, 1.7)	1.4 (1.2, 1.7)	2.5 (1.9, 3.2)
Bodymed BDMOXMTRBLK	3.5 (3.1, 3.9)	4.7 (3.7, 5.8)	6.0 (4.6, 7.7)	5.3 (4.3, 6.3)	2.8 (2.2, 3.5)	3.3 (2.7, 4.2)	3.3 (2.9, 3.8)	1.4 (1.1, 1.8)	2.5 (2.1, 3.0)	1.3 (1.0, 1.7)
Roscoe POX-ROS	4.3 (3.6, 5.1)	1.7 (1.5, 2.1)	2.3 (1.7, 3.3)	7.6 (4.7, 11.5)	2.1 (1.7, 2.9)	2.8 (2.1, 3.7)	9.9 (7.8, 12.3)	1.7 (1.4, 2.0)	1.9 (1.5, 2.4)	4.1 (3.2, 5.6)
CONTEC CMS50M	4.3 (3.8, 4.9)	6.1 (4.9, 7.5)	5.2 (3.8, 8.1)	7.5 (5.3, 10.5)	4.4 (3.4, 6.1)	3.4 (2.9, 4.0)	3.4 (2.6, 4.4)	3.1 (2.5, 4.5)	2.6 (2.1, 3.4)	2.9 (1.9, 4.4)
Biolight M70	4.5 (4.0, 5.0)	6.6 (5.7, 7.7)	6.2 (4.5, 8.5)	5.2 (3.8, 6.7)	3.2 (2.7, 4.2)	4.9 (3.5, 7.3)	3.8 (2.3, 5.9)	3.4 (2.7, 4.8)	3.6 (2.5, 5.1)	1.3 (1.1, 1.6)
**Lower LOA (95% CI, %)**										
Nellcor (Reference)	−2.3 (−2.5, −2.0)	−3.0 (−3.1, −2.5)	−3.1 (−3.8, −2.7)	−2.2 (−3.0, −2.1)	−1.5 (−2.1, −1.2)	−1.8 (−2.5, −1.1)	−0.6 (−0.9, −0.3)	−2.8 (−2.9, −1.9)	−1.5 (−1.8, −0.9)	−2.0 (−2.3, −1.5)
Nonin Onyx Vantage 9590	−2.2 (−3.9, −1.8)	−2.4 (−2.4, −2.2)	−2.0 (−2.0, −1.7)	0.0 (0.0, 0.3)	−2.9 (−2.9, −2.0)	−1.8 (−1.8, −1.2)	−1.0 (−1.0, −0.9)	−3.9 (−3.9, −2.9)	−9.6 (−9.6, −6.6)	−11.5 (−11.5, −1.6)
Masimo Mightysat	−2.4 (−3.1, −2.2)	−2.4 (−2.4, −2.3)	−2.5 (−2.5, −2.3)	1.1 (1.1, 1.7)	−1.5 (−1.5, −1.3)	−2.3 (−2.3, −1.8)	0.3 (0.3, 1.1)	−6.5 (−6.5, −3.1)	−3.3 (−3.4, −1.4)	−1.0 (−1.0, 0.4)
Walgreens MD300CN350R	−3.6 (−4.5, −2.7)	−4.5 (−4.5, −3.0)	−5.3 (−5.3, −4.5)	−0.2 (−0.2, 0.8)	−2.1 (−2.1, −1.6)	−2.7 (−2.7, −1.6)	0.9 (0.9, 1.1)	−3.8 (−3.8, −3.7)	−1.2 (−1.2, −1.1)	−12.5 (−12.5, −1.1)
Zacurate CMS 500DL	−2.7 (−6.0, −1.8)	−3.7 (−3.7, −0.1)	−7.9 (−7.9, −4.0)	1.6 (1.6, 2.4)	−0.1 (−0.1, 0.3)	−6.0 (−6.0, −1.5)	−0.1 (−0.1, 0.0)	−2.7 (−2.7, −2.1)	−1.3 (−1.3, −0.1)	−1.8 (−1.8, −1.3)
Walgreens OxyWatch C20	−2.4 (−5.0, −1.9)	−3.1 (−3.1, −2.4)	−5.0 (−5.0, −2.3)	−0.2 (−0.2, 0.1)	−3.0 (−3.0, −1.9)	−0.9 (−0.9, −0.8)	−0.1 (−0.1, 0.1)	−6.0 (−6.0, −3.5)	−1.9 (−1.9, −1.7)	−0.8 (−0.8, −0.5)
Choice MMed MD300CN340	−2.4 (−6.1, −1.3)	−2.8 (−2.8, −2.4)	−1.6 (−1.6, −0.3)	−1.3 (−1.3, −0.9)	−0.7 (−0.7, −0.3)	−0.6 (−0.6, −0.5)	−6.5 (−6.5, −6.1)	−4.5 (−4.5, −1.2)	−1.4 (−1.4, −1.2)	−7.2 (−7.2, −6.2)
Zacurate 500C	−9.1 (−15.9, −6.5)	−10.8 (−10.8, −8.9)	−8.0 (−8.0, −7.3)	−18.9 (−18.9, −15.9)	−2.5 (−2.5, −1.0)	−3.5 (−3.5, −2.9)	−5.7 (−5.7, −5.2)	−1.9 (−1.9, −1.7)	−2.6 (−4.0, −2.1)	−4.6 (−4.6, −2.9)
Bodymed BDMOXMTRBLK	−3.5 (−4.1, −2.5)	−8.7 (−8.7, −0.8)	−3.6 (−3.6, −2.3)	1.7 (1.7, 3.8)	−3.5 (−3.5, −1.4)	−4.1 (−4.1, −4.0)	1.0 (1.0, 1.8)	−3.1 (−3.1, −2.8)	−3.8 (−3.8, −1.3)	−1.4 (−1.4, −0.3)
Roscoe POX-ROS	−12.0 (−16.0, −7.5)	−3.7 (−3.7, −3.2)	−5.5 (−5.5, −3.3)	−10.2 (−10.2, −3.9)	−3.1 (−3.1, −2.6)	−8.9 (−8.9, −6.6)	−22.1 (−22.1, −21.3)	−3.2 (−3.2, −3.1)	−4.8 (−4.8, −4.1)	−12.0 (−12.0, −8.9)
CONTEC CMS50M	−11.0 (−12.4, −9.3)	−12.4 (−12.4, −10.3)	−11.0 (−11.0, −9.3)	−16.9 (−16.9, −11.9)	−11.8 (−11.8, −7.7)	−6.9 (−6.9, −6.6)	−6.7 (−6.7, −6.2)	−9.6 (−9.6, −3.6)	−8.0 (−8.0, −5.4)	−7.9 (−7.9, −7.2)
Biolight M70	−10.3 (−11.7, −9.2)	−11.7 (−11.7, −11.2)	−11.5 (−11.5, −8.7)	−4.7 (−4.7, −4.2)	−3.9 (−10.9, −3.5)	−6.5 (−6.5, −6.2)	−13.5 (−13.5, −10.3)	−12.2 (−12.2, −7.1)	−9.2 (−9.2, −8.0)	−0.4 (−0.4, −0.3)
**Upper LOA (95% CI, %)**										
Nellcor (Reference)	5.3 (4.9, 6.1)	3.5 (3.4, 5.0)	5.0 (3.8, 6.4)	8.0 (6.8, 8.1)	4.3 (3.7, 5.2)	6.7 (5.2, 12.6)	5.2 (4.2, 5.9)	3.4 (3.0, 3.8)	3.8 (3.2, 3.9)	3.8 (3.5, 4.9)
Nonin Onyx Vantage 9590	4.5 (4.1, 5.2)	2.4 (2.1, 3.9)	3.1 (2.9, 4.4)	4.3 (3.8, 4.6)	2.4 (1.0, 2.6)	5.2 (4.1, 5.8)	3.6 (2.2, 4.8)	3.0 (1.4, 3.1)	5.3 (5.2, 5.3)	1.7 (1.2, 1.7)
Masimo Mightysat	5.8 (4.8, 7.6)	3.5 (3.4, 3.5)	8.2 (7.7, 9.6)	6.9 (6.6, 6.9)	3.7 (2.4, 4.2)	4.0 (3.5, 4.8)	5.2 (4.9, 5.2)	2.5 (2.0, 3.0)	1.1 (1.0, 1.4)	3.8 (2.8, 6.1)
Walgreens MD300CN350R	5.7 (5.0, 7.2)	3.4 (2.8, 3.8)	5.0 (4.7, 6.5)	8.3 (7.8, 8.3)	3.3 (2.9, 3.5)	4.8 (2.7, 5.2)	6.0 (5.9, 6.0)	1.4 (1.0, 1.5)	4.1 (3.5, 4.3)	4.2 (3.6, 4.2)
Zacurate CMS 500DL	5.4 (4.7, 6.4)	5.7 (3.5, 5.7)	5.4 (4.0, 6.5)	6.4 (5.8, 6.4)	3.7 (3.5, 3.7)	4.3 (3.8, 4.8)	4.0 (3.4, 4.7)	1.9 (1.2, 2.5)	3.9 (3.7, 4.7)	3.0 (2.4, 3.8)
Walgreens OxyWatch C20	6.7 (4.0, 13.3)	4.6 (4.3, 4.6)	3.4 (3.1, 4.5)	14.1 (13.4, 14.1)	3.4 (2.8, 3.7)	4.2 (3.7, 4.8)	7.2 (6.7, 7.6)	1.8 (1.5, 3.0)	4.0 (2.5, 4.0)	2.2 (2.0, 2.7)
Choice MMed MD300CN340	6.2 (5.4, 7.4)	8.0 (7.4, 8.5)	4.9 (4.5, 6.0)	5.8 (5.3, 5.8)	6.2 (5.7, 6.2)	4.2 (3.9, 4.5)	4.2 (4.0, 4.2)	4.0 (3.8, 4.4)	1.8 (1.4, 1.9)	4.1 (3.4, 7.1)
Zacurate 500C	5.3 (4.7, 6.2)	5.0 (4.1, 5.5)	4.1 (3.4, 4.5)	9.8 (7.3, 9.8)	3.8 (3.4, 5.2)	3.1 (2.7, 3.5)	5.9 (5.3, 5.9)	3.0 (2.2, 3.5)	2.6 (1.1, 2.9)	4.5 (4.1, 4.6)
Bodymed BDMOXMTRBLK	8.5 (7.0, 11.1)	6.5 (6.1, 6.5)	11.7 (10.0, 12.6)	7.6 (6.4, 7.6)	5.5 (4.7, 5.5)	7.0 (5.7, 8.5)	4.7 (4.0, 5.2)	2.2 (1.6, 2.8)	4.8 (4.6, 5.4)	2.8 (2.4, 2.8)
Roscoe POX-ROS	6.7 (3.8, 13.7)	2.2 (1.6, 2.4)	3.5 (0.7, 6.7)	19.6 (14.4, 19.6)	3.8 (2.5, 6.6)	2.5 (1.9, 2.9)	12.7 (10.6, 13.7)	1.1 (0.7, 2.5)	4.8 (3.7, 5.0)	−0.3 (−0.6, −0.2)
CONTEC CMS50M	4.5 (3.9, 6.0)	3.0 (2.4, 4.5)	1.2 (−0.1, 19.3)	7.8 (2.1, 7.8)	4.4 (2.2, 5.7)	0.5 (−0.2, 0.5)	4.9 (3.9, 4.9)	5.0 (3.9, 5.5)	2.4 (0.9, 6.0)	3.1 (1.7, 3.2)
Biolight M70	10.0 (7.7, 13.4)	12.9 (11.4, 13.0)	14.0 (3.4, 14.8)	9.3 (8.9, 10.0)	5.5 (4.8, 8.7)	13.4 (4.0, 14.0)	2.9 (2.0, 3.4)	6.3 (3.9, 7.7)	4.1 (2.8, 4.2)	2.1 (1.7, 2.9)

Participants were studied using protocols previously described by our laboratory.^23^ Briefly, inspired oxygen, nitrogen, and carbon dioxide partial pressures were monitored in real time and adjusted via a partial rebreathing circuit to achieve stable target arterial oxygen saturation (SaO 2) plateaus between 70% and 100% and PaCO2 values of 35–45 mm Hg. Participants were motionless and each desaturation lasted approximately 20 min. To determine stability of the plateaus, judgement of the investigator controlling inspired gas mixtures as well as a software algorithm were used. The algorithm continuously measures the slope of calculated oxygen saturation (ScO2) based on end tidal oxygen concentration as well as the measured SpO 2 on the clinical reference oximeter. The slope is converted to the amount that oxygen saturation would change over 1 min given the current slope. If this change is predicted to be ≥ 1.5, then a red light appears on the study computer and sampling is not done. In accordance with regulatory guidance, we waited for at least a period of at least 60 s between plateaus, and 30 s between samples within a plateau. Time to plateau stability ranged from 1 to 5 min. There is currently no standard approach for defining stability for controlled desaturation studies. Pulse oximeter readings were recorded each time an arterial blood sample was taken. Clinical research coordinators who were recording SpO2 values on the devices under test were blinded to arterial SaO2. Each device was tested on at least four independent finger locations chosen at random. We compared POX readings (SpO2) with SaO2 (Radiometer ABL90 Flex Plus) to calculate the error (SpO2–SaO2), the bias (mean of the error), the precision (SD of the error), and the root mean square error (ARMS).

Skin pigmentation of each participant was measured subjectively by pFP and objectively with a spectrophotometer (Konica Minolta CM-700d) according to our previously published protocol.^23^ Perceived Fitzpatrick was assigned by study personnel by matching forehead colour to a visual analogue Fitzpatrick Scale obtained online^23^ and printed with a colour laser printer (Sharp MX-M363N). The spectrophotometer with 3 mm aperture was utilised to measure the relative intensity of reflected light from 400 to 700 nm with 10 nm resolution and specular reflection was included. Measurements were obtained in triplicate at nine anatomical sites in each participant: middle of the fingernail, dorsal distal phalanx (DDP) between the joint and the fingernail, palmar distal phalanx (PDP), inner upper arm, front and back of the earlobe, cheek, forehead, and the external surface of one nare.^23^ All measurements, including pFP, were taken with ambient fluorescent overhead lighting conditions in the laboratory. The CM-SA Skin Analysis Software (Konica Minolta, Tokyo, Japan) was used to process measured CM-700d data with the illuminant set to ‘D65’ and observer angle set to 10° before exporting CIELAB L∗,a∗,b∗ colour space coordinates. Conversion from LAB values to RGB values was achieved using the *lab2rgb*() function in MATLAB, or the python-colormath package (3.0.0) in Python, with D65 white point and sRGB colorspace. Individual typology angle (ITA) has been shown to correlate with melanin content and is defined as the angle in the L∗b∗ coordinate plane between (0,50) and (b∗,L∗) by the equation.

ITA = [arctan((L∗-50)/b∗)] ∗180/π.^25^, ^26^, ^27^

For analysis, participants were grouped by ITA values measured at the DDP into upper, middle and lower thirds, corresponding to lightest, medium and darkest pigmentation respectively. The ITA cut-off values that resulted in near equal groupings for lightest, medium, and darkest pigmentation were ITA > 30° (light), 2° < ITA<30° (medium), and ITA < 2° (dark). With respect to categorising participants by pFP, in accordance with other studies^28^ light pigmentation was defined as pFP I/II, medium was defined as pFP III/IV, and dark pigmentation was defined as pFP V/VI.

### Ethics

This study was approved by the University of California, San Francisco (UCSF) Committee on Human Research IRB. It is consistent with Standards for Reporting Diagnostic Accuracy Studies.^29^ Written informed consent was obtained from all participants (UCSF IRB #21-35637) in accordance with the Declaration of Helsinki.

### Statistics

All analyses were performed using MatLab (The Mathworks, Inc., Boston, MA) and STATA v 17.0 (Statacorp., College Station, TX). The Shapiro–Wilk test was used to assess normality. Bias was computed as the mean difference between the oximeter SpO 2 and the corresponding arterial SaO 2, and absolute bias was calculated as the mean of the absolute difference. Given that hemoximeters at times report SaO2 values > 100%, and it is uncertain how pulse oximeter software handles values where the algorithm suggests >100% SpO2 or how values are truncated, data points where SaO2 and/or SpO2 were ≥100% were excluded from analysis to avoid the potential problem of incorrectly altering the mean bias values. Data were visualised using a modified Bland-Altman plot for each tested device where error (SpO2-SaO2) is plotted against SaO2. Giving non-normally distributed error (by Shapiro–Wilk), non-parametric limits of agreement were determined by determining the 2.5% and 97.5% percentiles of the ordered errors.^30^

Linear regression was performed between ITA values measured at different anatomic locations in the same individual across all 34 participants and Spearman rank correlation coefficients were determined for each ITA comparison between anatomic sites. A Fischer transform was used to determine 95% confidence intervals for Spearman rank correlation coefficients.

Average root mean square error (ARMS) was calculated as the square root of the mean value of the squared SpO 2-SaO 2 differences. The 95% confidence intervals for all values were calculated by using the *bootci* function in MatLab to bootstrap with random resampling over 5000 repetitions using the bias corrected and accelerated percentile method.

Spectrophotometer measurements were made in triplicate at each skin site with the spectrophotometer held in a single position. LAB and ITA values were derived from spectrophotometer measurements, and the median ITA was used for analysis to minimise impact of outlier values. Standard deviation of ITA for each individual at each anatomic site from the triplicate readings was determined to assess measurement reproducibility.

### Role of funders

Funders did not have any role in study design, data collection, data analyses, interpretation, or writing of this report.

## Results

Readings from 11 fingertip POXs and the clinical reference POX were obtained, corresponding to 4360 blood samples in 34 participants. Each POX tested had >200 samples of coupled SpO 2 and SaO 2 data from at least ten participants. All devices had at least two participants with dark pigmentation (exceeding 15% of the study population) as per current FDA Guidance. Demographics of all study participants are presented in Table 1.

**Fig. 1:**
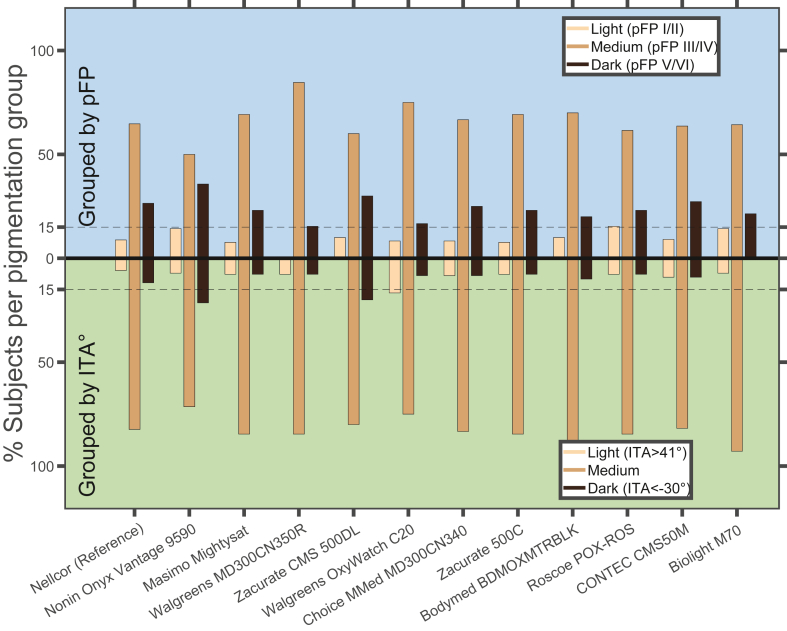
**Comparison of participants grouped by objective and subjective measures**. The pFP and ITA range of healthy volunteers studied per device. Upper Y axis shows percent of participants per device grouped by pFP with pFP I/II defining the light pigmentation group, pFP III/IV defining the medium pigmentation group, and pFP V/VI defining the dark pigmentation group. Lower Y axis shows percent of participants per device grouped by ITA, with ITA > 41° defining the light pigmentation group, −30° < ITA < 41° defining the medium pigmentation group, and ITA < −30° defining the dark pigmentation group. Current FDA guidelines require a minimum of 15% of participants in a performance study to have dark pigmentation (horizontal dashed line).

Whether an individual met criteria to be included in the ‘darkly-pigmented’ group depended on whether pigment was assessed by subjective or objective means. Fewer participants were categorised as having dark skin pigment when using the objective cutoff of ITA < −30° as compared to the subjective pFP classification of pFP V/VI. When using ITA, nine of 11 POXs tested as well as the clinical reference POX did not have enough data to meet the FDA threshold for proportion of participants with dark pigment compared to all POXs meeting FDA threshold when assessed with pFP (Fig. 1).

**Fig. 2:**
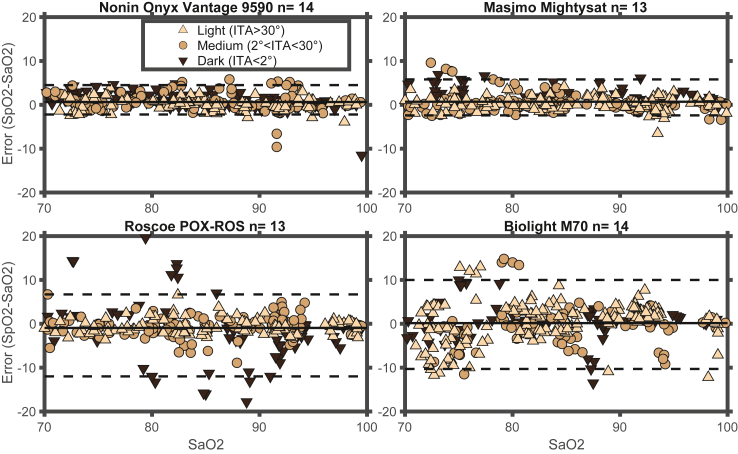
**Bland-Altman plot of best and worst performing devices showing error vs SaO 2**. Error (SpO 2–SaO2) vs SaO2 for the two best performing (lowest ARMS) and two poorly performing devices. Error (pulse oximeter oxygen saturation [SpO2] − arterial blood oxygen saturation [SaO2) is plotted against SaO2 measured by an ABL90 hemoximeter (Radiometer). For each device, data points are grouped by pigmentation (lightest third, medium third, and darkest third) as determined by ITA measured at the DDP. Dashed horizontal lines are the upper and lower nonparametric limits of agreement, and horizontal solid line is the bias (mean of the error).

There was a wide range in SpO 2-SaO 2 error between devices, and the two best (Nonin and Masimo) as well as two poorly performing devices (Biolight and Roscoe) are highlighted in Fig. 2, where the device error (SpO2–SaO2) is plotted against SaO2. Of note because there were few participants in the traditionally defined ITA < −30° ‘darkly pigmented’ group, we applied alternative ITA cutoffs that divided participants into lightest pigmented third (11 participants, DDP ITA 30.1°–46.8°), medium pigmented third (13 participants, DDP ITA 13.7°–28.0°), and darkest pigmented third (ten participants DDP ITA -67.0°–1.8°). In Supplemental Figures S1 and S2, similar plots are presented for the remaining devices.

**Fig. 3:**
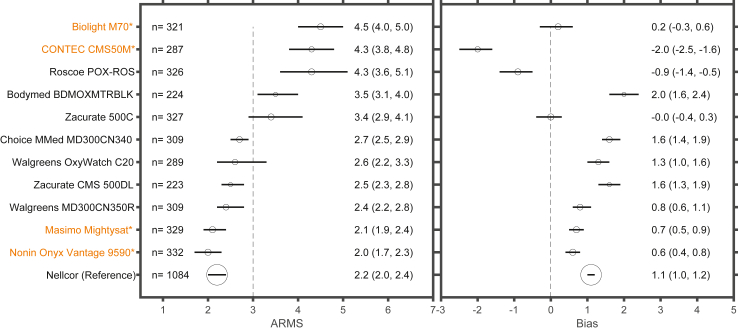
**Forest plot of ARMS and bias across SaO 2 values of 70–100%**. Forest plot showing ARMS and bias for each device (lines show 95% confidence interval). Within each device the ARMS and bias are determined among SaO2 values between 70 and 100%. The dashed vertical line represents an ARMS of 3%. The size of the circles in each line is proportional to the number of measured data points. POX with 510k compliance are highlighted in orange and with an asterisk.

**Fig. 4:**
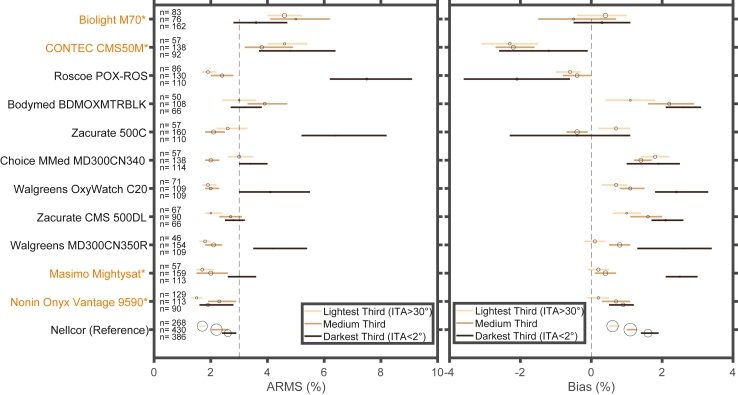
**Forest plot of ARMS and bias for each device among different skin colours**. Forest plot showing ARMS and bias for each device (lines show 95% confidence interval). Within each device the ARMS and Bias are determined among participants with the lightest, medium, and darkest pigmentation (defined by ordering participants by DDP ITA and selecting ITA cutoffs that create equal groups). For each device, n represents the number of samples among the light, medium, and darkly pigmented groups, and the size of the circles within each line is proportional to n. The dashed vertical line represents an ARMS of 3%. POX with 510k compliance are highlighted in orange and with an asterisk.

The ARMS, bias, absolute bias, precision, and non-parametric limits of agreement for each device, including all data from 70 to 100% as well as grouping data by SaO 2 deciles (70–80%, 80–90%, 90–100%) and pigmentation groups (lightest third, middle third, and darkest third) are presented in Table 2. Six of the 11 POXs tested and the clinical reference POX met the FDA criteria of ARMS ≤3% between the SaO2 range of 70–100%, while the remaining five POX devices, including 2 with 510(k) premarket notification clearance, had an ARMS >3% (Fig. 3, Table 2). Eight of the 11 POXs and the clinical reference POX met ISO Standard criteria for ARMS ≤4%. Ten of 11 devices and the reference device demonstrated higher ARMS in study individuals categorised at the lowest third of ITA values (dark), compared with the highest third of ITA values (light), measured at the DDP (Fig. 4). Seven of the 11 POXs tested and the reference POX demonstrated a positive bias among individuals with darker pigmentation compared to individuals with lighter pigmentation (Fig. 4). Most POX demonstrated higher ARMS at lower SaO2 values, especially in individuals with darker skin pigment (Table 2, Supplemental Figures S3 and S4).

**Fig. 5:**
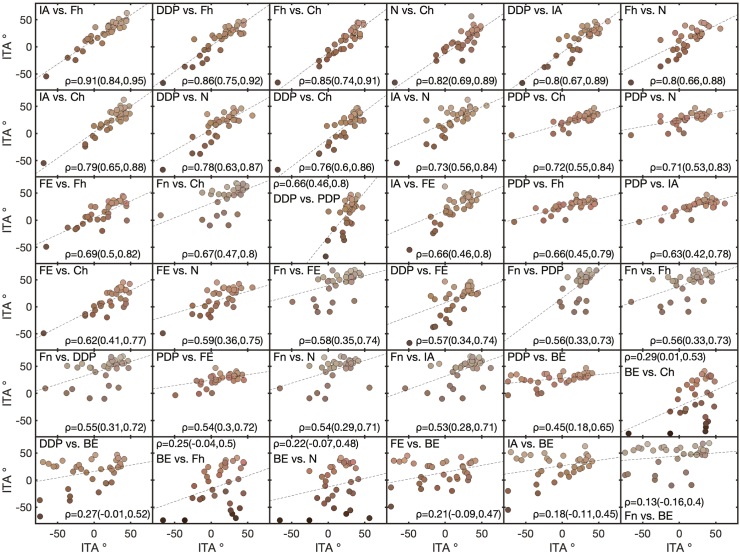
**Correlation between individual typology angle measurements at different anatomical sites**. Correlation between ITA measurements at different anatomical sites. Each box plots the correlation of ITA measurement at two different anatomical sites. Colour used to represent data points is intended to represent study participant skin colour based on LAB values when transformed to RGB. Spearman rank correlation coefficient ⍴ with 95% confidence intervals is provided in each plot. Reproduced print and online figures may not portray accurate colour as intended. (Fn = fingernail, DDP = dorsal distal phalanx, IA = inner upper arm, FE = front earlobe, BE = back earlobe, N = nare, Ch = cheek, PDP = palmar distal phalanx, Fh = forehead).

While ITA values measured at different anatomic sites within the same participant showed considerable variation, some sites like the inner arm, forehead, and DDP showed strong correlation (Fig. 5). The standard deviation of triplicate ITA measurements within the same participant (Supplemental Figure S5) did not exceed 10° when measuring the inner arm, cheek, forehead, or DDP. However, for the remaining anatomic sites, a higher degree of variability among triplicate measurements was noted with ITA standard deviation exceeding 10°. The back earlobe showed the greatest degree of variability (Supplemental Figure S5), and also demonstrated the lowest Spearman ⍴ coefficients when correlated to other anatomic sites (Fig. 5).

**Fig. 6:**
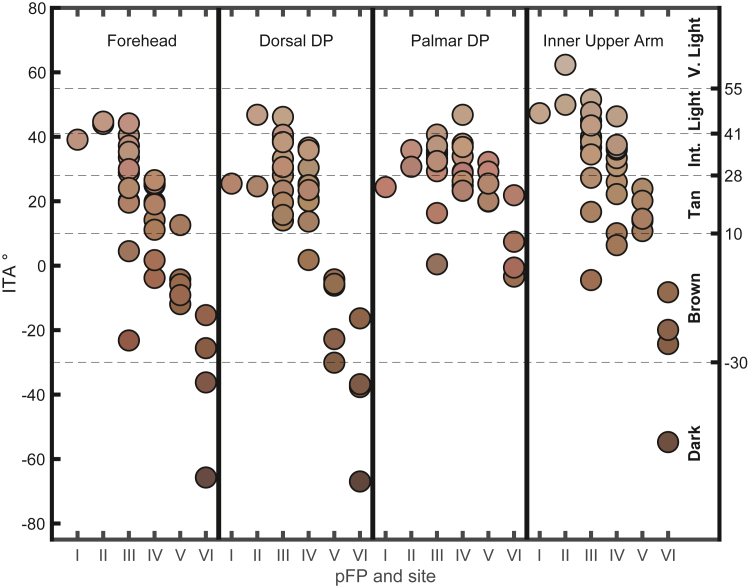
**Participant individual typology angle at four anatomical sites, compared to perceived Fitzpatrick Scale**. ITA values from individual participants grouped by pFP at four anatomical sites (forehead, DDP, PDP, and inner upper arm). Each data point is the median ITA value from a single individual with given pFP at a given anatomic site. Colour used to represent data points is intended to represent study participant skin colour based on LAB values when transformed to RGB. Dotted horizontal lines represent ITA cutoffs published by Del Bino et al., 2013 for skin phenotype (very light >55° > light > 41° > intermediate >28° > tan >10° > brown > −30° > dark).^25^ The colour of each data point represents study participant skin colour measured by colorimetry but may not accurately portray perceived colour by a human observer.

The range of ITA values observed among participants with the same pFP varied based on anatomic site (Supplemental Table S1). For example, for individuals with pFP I-VI, ITA values measured at the fingernail (−9.8° to 68.1°) and PDP (−3.4 to 40.7) showed a narrower range of values and lighter pigmentation (higher ITA) than ITA values measured at the forehead (−65.7° to 44.7°) (Fig. 6, Supplement Figures S6 and S7). Fig. 6 and Supplement Figures S6 and S7 demonstrate an overall decrease in ITA with increasing pFP across multiple sites (i.e. forehead ITA −23.2° to 44.7° for pFP I/II/III and −65.7° to −15.3° for pFP VI). However, among participants with the same pFP of III, forehead ITA values ranged across 4 separate ITA-defined pigmentation groups (Del Bino-defined Brown (−30° to 10°), Tan (10°–28°), Intermediate (28°–41°), and Light (41°–55°). A visual representation of the forehead skin colour of study participants with corresponding ITA values is presented in Supplemental Figure S8.

## Discussion

This study evaluated the performance of 11 fingertip POXs and found that six devices had ARMS ≤3% (FDA 510k compliance) and eight devices had ARMS ≤4% (ISO Standard compliance). Of the four POXs that had received FDA 510(k) compliance, only two demonstrated an ARMS ≤3% in our study. We found that the pFP scale was inadequate to assess skin pigmentation, whereas objective skin pigmentation measurement by ITA was feasible and has potential to better ensure enrolment of individuals spanning the full spectrum of skin pigmentation. These findings may be especially relevant in settings that care for populations with dark skin pigmentation and where low-cost fingertip POXs are commonly used.

Our findings corroborate prior studies that have demonstrated low-cost fingertip POXs have variable performance, which is at times inconsistent with manufacturers’ regulatory claims.^29^ We found that some POXs exhibited poor performance on participants with darker skin pigmentation, though note that some prior studies such as Bothma et al. have reported no effect of skin pigment on device performance; however, our study tested oximeters at lower SaO 2 values and with more thoroughly characterised skin pigment.^8^

*Our data show that current regulatory guidance and standards for POXs do not adequately ensure diversity of skin pigmentation.* At the time of this study, FDA guidelines mandate that POXs be tested on a minimum of ten participants with at least two, or 15% of participants (whichever is larger) being ‘darkly pigmented’.^31^^,^^32^ There are two problems with this guidance: first, the small absolute number of individuals with dark skin; and second, the lack of specific requirements on how to assess skin pigment.

*Lack of regulatory guidance or scientific consensus on how to measure skin colour and how to define dark and light skin phenotypes is a challenge that must be addressed to ensure equitable device performance when skin colour is a factor.* Several prior studies have commonly used race as a surrogate for skin colour and assumed that inclusion of certain races would ensure appropriate inclusion of darkly pigmented people. Data from many countries show that the range of skin colours within most ethnic or racial categories is as wide as between them, refuting the use of race as a surrogate for skin colour or the use of skin colour as a surrogate for race.^33^^,^^34^ There are limited data to guide optimal use of ITA including which numerical cutoffs to use for categorising skin pigment (i.e. light vs dark). The ITA was introduced in 1991 with suggested ITA cutoffs to categorise skin colour (very light >55° > light >41° > intermediate >28° > tan >10° > brown). However, that study included only Caucasian individuals and only non-sun-exposed skin. Subsequent work by Del Bino et al., in 2013 included a more diverse study population and introduced an additional cut-off of < −30° to denote dark pigmentation^25^ Notably, this expanded categorization by ITA was still based on arguably flawed bins from the original 1991 data and kept bin cutoffs unchanged despite measuring ITA at a sun-exposed site. In our study, for the purpose of comparison with the pFP scale, we considered defining dark pigment as an ITA < -30°. However, given the small number of participants with ITA < −30° and uncertainty around the validity of that cutoff, we conducted our analysis by grouping participants into upper, middle, and lower thirds of ITA values found in our study pool (lightest, medium, darkest). In our study, all device testing cohorts met current minimum FDA criteria with respect to inclusion of individuals with ‘darkly pigmented’ skin despite the fact that most of the participants in our study did not have objectively dark pigmentation (ITA < −30°). This highlights the fact that when ‘dark pigmentation’ is not objectively defined, there is a risk that one can meet regulatory requirements while failing to adequately test people across the full range of skin pigmentation. As illustrated by Fig. 1, when subjectively categorising participants as ‘dark’ using the pFP V/VI categories, we are led to the false conclusion that the study cohort has adequate inclusion of darkly pigmented individuals. However, when objectively categorising dark skin pigment using Del Bino's ITA < −30° cutoff, the same cohort demonstrated inadequate inclusion of darkly pigmented individuals (Fig. 1). Efforts are currently underway by multiple initiatives to better define methods for assessing skin pigmentation.

*Our data support several steps that could be taken now to update regulatory guidance and improve performance standards.* The commonplace reliance on subjective, non-standardised skin pigmentation assessment tools (e.g. pFP) is problematic and should be abandoned for several reasons, including the lack of standardised colours, inter-operator variability and bias, and the misappropriation of the initial purpose of the scales. For each subjectively assessed pFP phototype, we found a wide and overlapping range of objectively measured ITA values (Fig. 6, Supplemental Figures S6 and S7). A key problem with scales like pFP is the lack of standardised colours (i.e. colours are not traceable to a specific RGB) and consequent gross variability in visual appearance of published scales. The flaws highlighted in the use of the pFP are likely similarly seen in other subjective non-standardised scales, such as the Massey-Martin Scale.^23^ Subjective scales that include standardised colours (e.g. the Monk Skin Tone Scale^23^), may address some limitations of existing subjective colour scales, however, objective skin pigment assessment (e.g. ITA) may provide a path for avoiding the limitations above while also ensuring traceability and comparability of data as well as avoiding potential operator bias seen with subjective scales.

*The optimal site for objective measurement of skin pigment has not been established but is an additional important consideration when defining skin pigment diversity.* Skin pigment varies across the body (Fig. 6, Supplemental Figures S6 and S7) due to natural distribution of pigment, sun exposure, and practical challenges of capturing data using spectrophotometry. For example, the back of the earlobe is challenging due to its small size and the fingernail is challenging due to its rigid, convex surface. The optimal site for skin colour measurement for the purpose of POX performance is uncertain though likely is one that directly impacts the physio-optical properties where the POX is placed. In theory, an appropriately placed POX probe on the finger aligns the LED emitter and sensor through the fingernail and the PDP. However, due to scattering and reflection of light throughout the finger, it is likely that the DDP is relevant and impacts POX performance.^35^ Given the strong correlation of the DDP and forehead ITA (Spearman ⍴ = 0.86), it is likely that study recruitment based on forehead colour can achieve a similar range of DDP ITA values.

*Another finding highlighted by the present study which contributes to variable performance of POXs available on the market is the lack of harmonisation among regulatory agency requirements.* While FDA guidelines mandate ARMS ≤3% for transmittance devices to reach 510(k) compliance, current ISO Standards mandate an ARMS ≤4%. Given that all POXs can be expected to perform worse in real-world, patients with an illness (as compared to controlled laboratory studies in healthy volunteers),^19^ and given ongoing concerns around additional errors in people with darkly pigmented skin, an ARMS ≤4% is likely too low of a bar to optimise patient safety and health equity. Harmonisation of global standards to an ARMS ≤3% could help eliminate multiple coexisting standards while also raising the bar for POX performance.

### Caveats and limitations

This study has several limitations. First, we enrolled only young, healthy adults which do not reflect the heterogeneity of anatomy and pathophysiology of patients with an illness, children or people with comorbidities (e.g. hypercholesterolemia, hypertension, and obesity). Additionally, we did not account for perfusion (i.e. pulsatility amplitude), a factor known to impact POX performance (which is not adequately accounted for in ISO standards or FDA guidance).^20^ Here we tested relatively few POXs, and the applicability of our findings to other POXs (including other brands, models, or production years) is uncertain. There are also several additional limitations in the study design including the relatively small sample size as well as lack of regulatory guidance on the use of limits of agreement in Bland–Altman analyses for nonparametric data. The sample size is based on existing FDA and ISO recommendations, not our own sample size calculations or analytical design. Due to limited statistical power, we were ultimately unable to fully assess the impact of skin pigmentation on POX performance. Further work is underway to clarify optimal sample size for better characterising impact of skin colour on device performance. An additional limitation relates to ITA and the prior discussion of data supporting its use. While ITA is a reproducible, quantitative surrogate for pigmentation, it is not a perfect estimate of melanin content or colour.^36^ A final limitation of this and similar studies, is the low number of individuals recruited with dark skin. This reflects the distribution of skin pigmentation of the population residing within the city where the study was conducted as well as previously described challenges of recruiting participants with diverse skin colours.^37^ Prioritising and addressing these challenges are essential components to improving equity in the performance of medical devices.

### Conclusion

Fingertip POXs have variable performance, frequently not meeting regulatory requirements for clinical use, and at times contradicting claims made by manufacturers. The FDA guidance and ISO standards at the time of this study do not adequately account for the potential impact of skin pigmentation on POX performance. We recommend that the pFP scale and other non-standardised, subjective skin colour scales no longer be used for defining diversity of skin pigmentation in POX validation studies. Further studies are needed to better define methods for ensuring diversity to improve equitable performance of POXs.

## Contributors

Dr Gregory Leeb: conceptualisation, data analysis, data collection, data curation, investigation, methodology, data interpretation, writing–original draft and editing.

Dr Isabella Auchus: conceptualisation, data collection, data interpretation, data curation, investigation, methodology, writing -original draft and editing.

Dr Leonid Shmuylovich: conceptualisation, data curation, methodology, validation, data analysis, data interpretation, figures, writing–original draft, reviewing & editing.

Dr Philip Bickler: conceptualisation, data collection, data analysis, data curation, methodology, data interpretation, writing -reviewing & editing.

Dr John Feiner: data collection, data analysis, data interpretation, figures, writing–original draft, reviewing & editing.

Kelvin Moore Jr.: writing–original draft and reviewing & editing.

Jana Fernandez: data collection, writing–reviewing & editing.

Yu Celine Chou: data collection, writing–reviewing & editing.

Deleree Schornack: data collection, writing–reviewing & editing.

Caroline Hughes: data collection, writing–reviewing & editing.

Dr Tyler Law: conceptualisation, data collection, data analysis, data interpretation, figures, writing–reviewing & editing.

Professor Ellis Monk: data interpretation, writing–original draft, reviewing & editing.

Dr Elizabeth Igaga: data interpretation, writing–original draft, reviewing & editing.

Dr Olobunmi Okunlola: data interpretation, writing–original draft, reviewing & editing.

Dr Shamsudini Hashi: writing–reviewing & editing.

Michael Bernstein: conceptualisation, writing–reviewing & editing.

Dr Michael Lipnick: conceptualisation, data collection, data interpretation, supervision, funding acquisition, investigation, methodology, writing–original draft, reviewing & editing.

Dr Jenna Lester: writing–reviewing & editing.

All authors read and approved the final version of the manuscript.

Dr Leonid Shmuylovich and Dr John Feiner have accessed and verified the underlying data.

## Data sharing statement

Data are available from the authors upon reasonable request and with the permission of the institution.

## Declaration of interests

Michael Bernstein receives consulting fees from University of California, San Francisco.

No other authors have any interests to declare, as per the International Committee of Medical Journal Editors disclosure of interests form.

## Appendix A Supplementary data

Supplemental Figure S1 **Bland-Altman plot of four devices showing error vs SaO2**. Error (SpO2 - SaO2) vs SaO2 for four of the POX tested, including the Nellcor tabletop clinical reference monitor. Error (pulse oximeter oxygen saturation [SpO2] − arterial blood oxygen saturation [SaO2]) is plotted against SaO2 measured by an ABL90 hemoximeter (Radiometer). For each device, data points are grouped by pigmentation (lightest third, medium third, and darkest third) as determined by ITA measured at the dorsal distal phalanx. Dashed horizontal lines are the upper and lower nonparametric limits of agreement, and horizontal solid line is the bias (mean of the error).

Supplemental Figure S2 **Bland-Altman plot of four devices showing error vs SaO2**. Error (SpO2 - SaO2) vs SaO2 for four of the POX. Error (pulse oximeter oxygen saturation [SpO2] − arterial blood oxygen saturation [SaO2]) is plotted against SaO2 measured by an ABL90 hemoximeter (Radiometer). For each device, data points are grouped by pigmentation (lightest third, medium third, and darkest third) as determined by ITA measured at the dorsal distal phalanx. Dashed horizontal lines are the upper and lower nonparametric limits of agreement, and horizontal solid line is the bias (mean of the error).

Supplemental Figure S3 **Forest plot of ARMS and bias across difference SaO2 ranges**. Forest plot showing ARMS and Bias for each device (lines show 95% confidence interval). Within each device the ARMS and bias are determined among ranges of SaO2 values: 70-80%, 80-90%, 90-100%. The dashed vertical line represents an ARMS of 3%. The size of the circles within each line is proportional to the number of measurements.

Supplemental Figure S4 **Pulse oximeter performance (ARMS) by SaO2 decile and individual typology angle pigmentation ranges**. For each device tested, subject data are grouped by SaO2 decile and ITA pigmentation ranges including lightest pigmented third of subjects (ITA >30°), medium pigmented third of subjects (30°>ITA>2°), and darkest pigmented third (ITA<2°), and ARMS is calculated for each device per decile and ITA pigmentation range. Each circle is the ARMS for a single device at a given SaO2 decile and ITA pigmentation range. Circles connected by lines represent ARMS values from the same device at a given SaO2 decile with varying ITA pigmentation range.

Supplemental Figure S5 **Standard deviation of individual typology angle values captured across anatomical sites.** Standard deviation between the three repeated ITA measurements shown by site. Colour used to represent data points is intended to represent study subject skin colour based on LAB values when transformed to RGB. The colour of each data point represents study subject skin colour measured by colorimetry but may not accurately portray perceived colour by a human observer.

Supplemental Figure S6 **Perceived Fitzpatrick Scale compared with individual typology angle measured at four anatomical sites**. Inter- and intra-subject range of ITA values compared to pFP. ITA measured at four anatomical sites (fingernail, dorsal distal phalanx, palmar distal phalanx, back earlobe) compared to pFP for all subjects. Each vertical column of data points represents one subject. Colour used to represent data points is intended to represent study subject skin colour based on LAB values when transformed to RGB. Horizontal lines represent ITA cutoffs published by Del Bino et al. 2013 for skin phenotype (very light > 55° > light > 41° > intermediate > 28° > tan >10° > brown > −30° > dark) ^25^. Reproduced print and online figures may not portray accurate colour as intended.

Supplemental Figure S7 **Perceived Fitzpatrick Scale compared with individual typology angle measured at five anatomical sites**. Inter- and intra-subject range of ITA values compared to pFP. ITA measured at five anatomical sites (inner upper arm, front earlobe, forehead, nare, cheek) compared to pFP for all subjects. Each vertical column of data points represents one subject. Colour used to represent data points is intended to represent study subject skin colour based on LAB values when transformed to RGB. Horizontal lines represent ITA cutoffs published by Del Bino et al. 2013 for skin phenotype (very light > 55° > light > 41° > intermediate > 28° > tan >10° > brown > −30° > dark)^25^. The colour of each data point represents study subject skin colour measured by colorimetry but may not accurately portray perceived colour by a human observer.

Supplemental Figure S8 **Visualisation of forehead skin colour of study subjects**. Forehead skin colour of study subjects by ITA and calculated RGB for each device tested. Skin colour represented in this figure is based on LAB values that are derived by the reflectance curves measured by the spectrophotometer, and the LAB values were transformed to RGB. The colour of each data point represents study subject skin colour measured by colorimetry but may not accurately portray perceived colour by a human observer.

Supplemental Table S1 ITA range for each pFP.
