# Supplementary figures and images for: The performance of 11 fingertip pulse oximeters during hypoxemia in healthy human participants with varied, quantified skin pigment
Source: eBioMedicine. 2024 Mar 8;102:105051. doi: 10.1016/j.ebiom.2024.105051 (PMC10943300; doi:10.1016/j.ebiom.2024.105051)

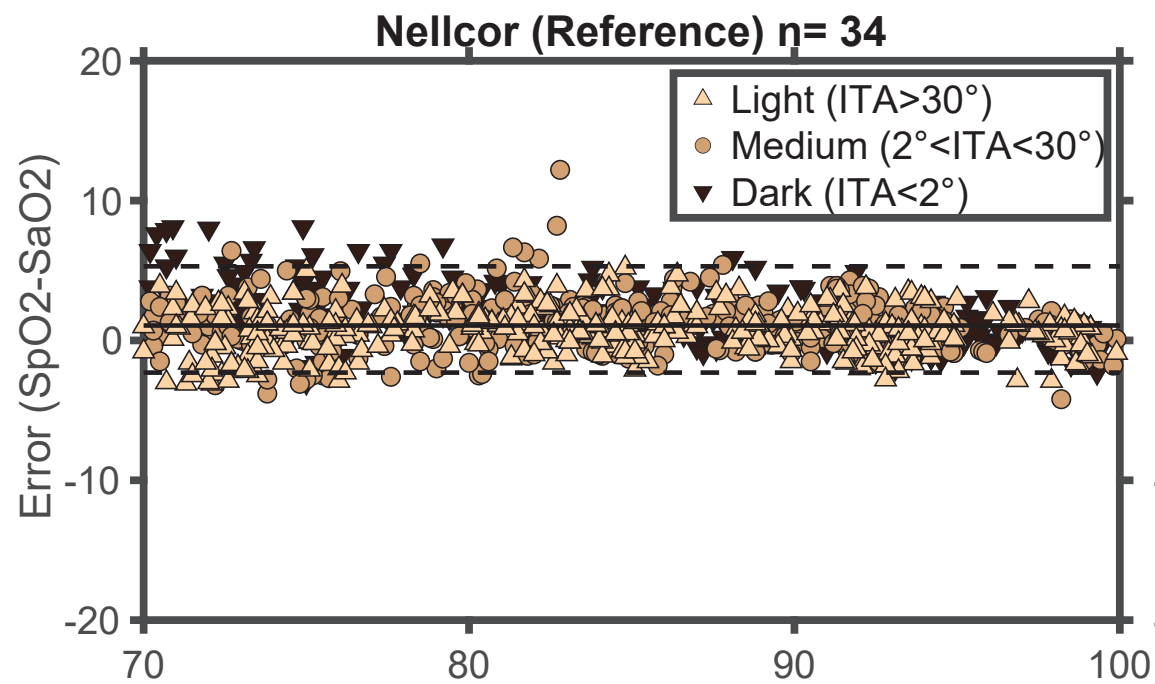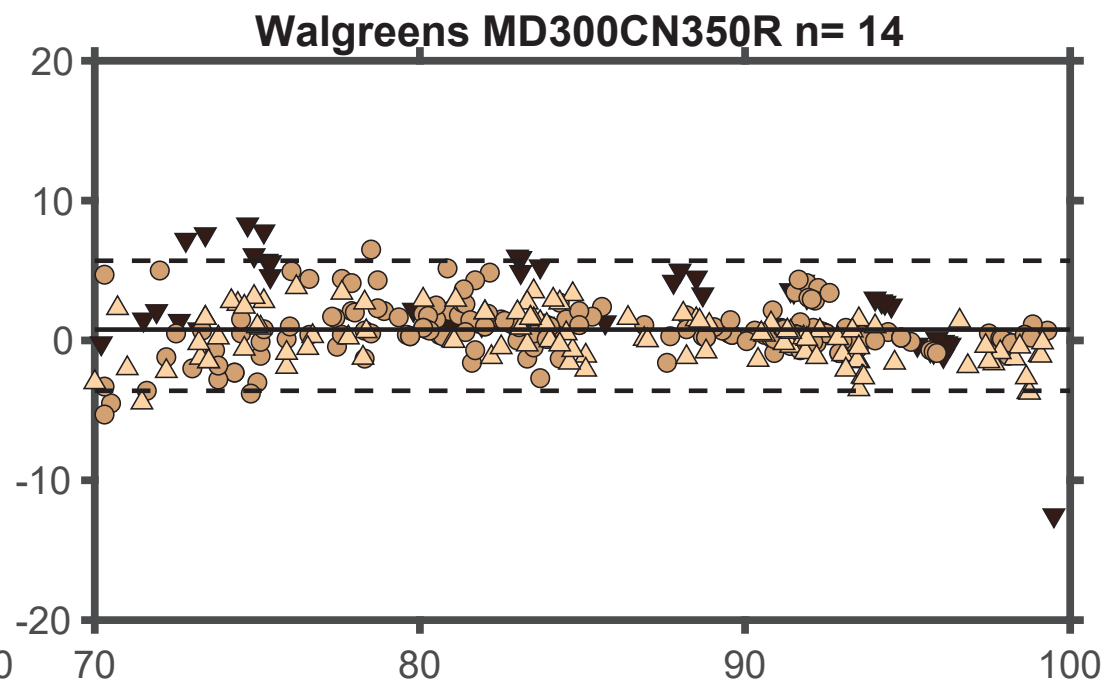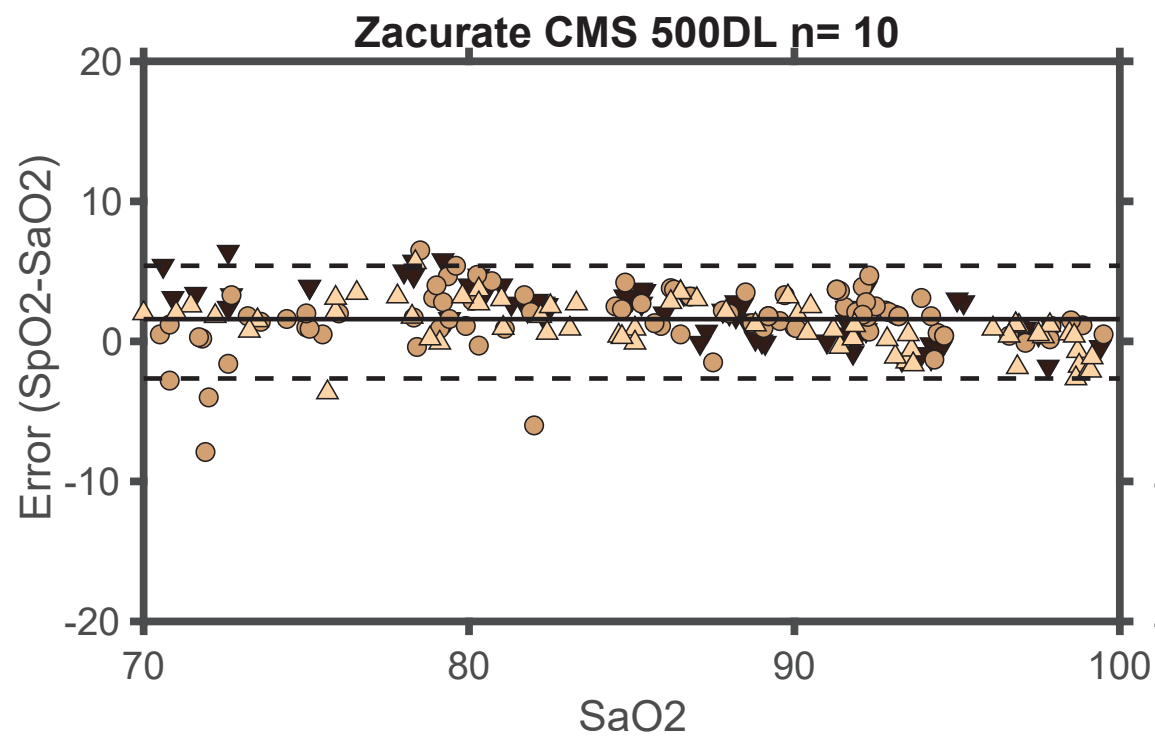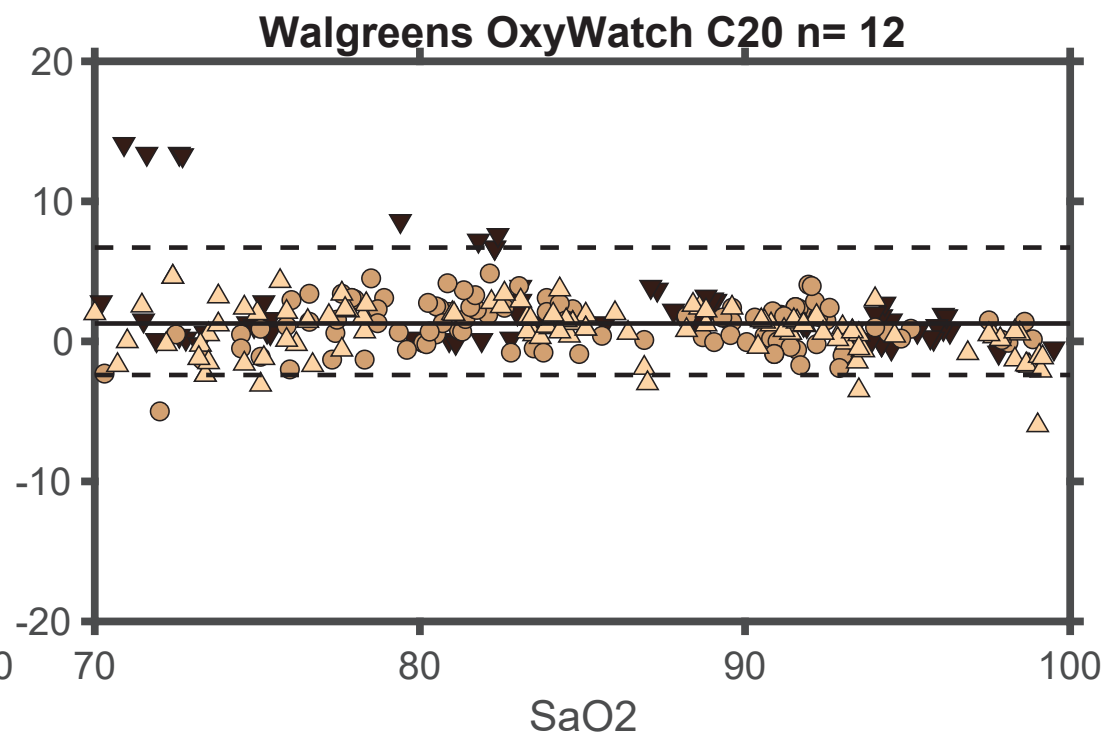

Supplement: Supplemental Figure S1 — Bland-Altman plot of four devices showing error vs SaO2. Error (SpO2 - SaO2) vs SaO2 for four of the POX tested, including the Nellcor tabletop clinical reference monitor. Error (pulse oximeter oxygen saturation [SpO2] − arterial blood oxygen saturation [SaO2]) is plotted against SaO2 measured by an ABL90 hemoximeter (Radiometer). For each device, data points are grouped by pigmentation (lightest third, medium third, and darkest third) as determined by ITA measured at the dorsal distal phalanx. Dashed horizontal lines are the upper and lower nonparametric limits of agreement, and horizontal solid line is the bias (mean of the error). [file mmc1.pdf]

**Choice MMed MD300CN340 n= 12**

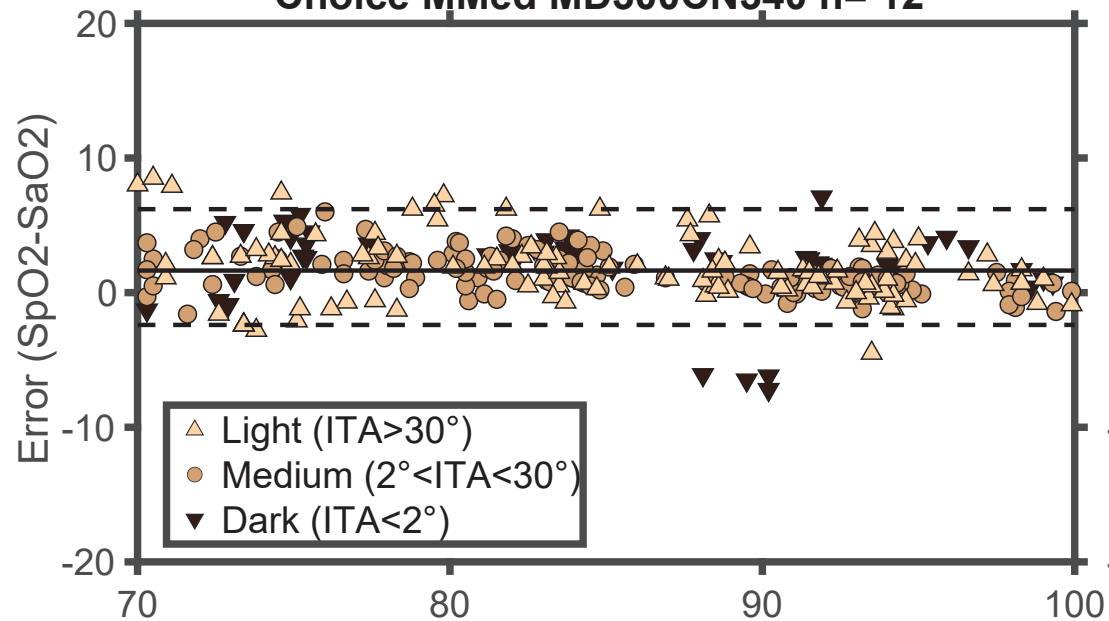

**Zacurate 500C n= 13**

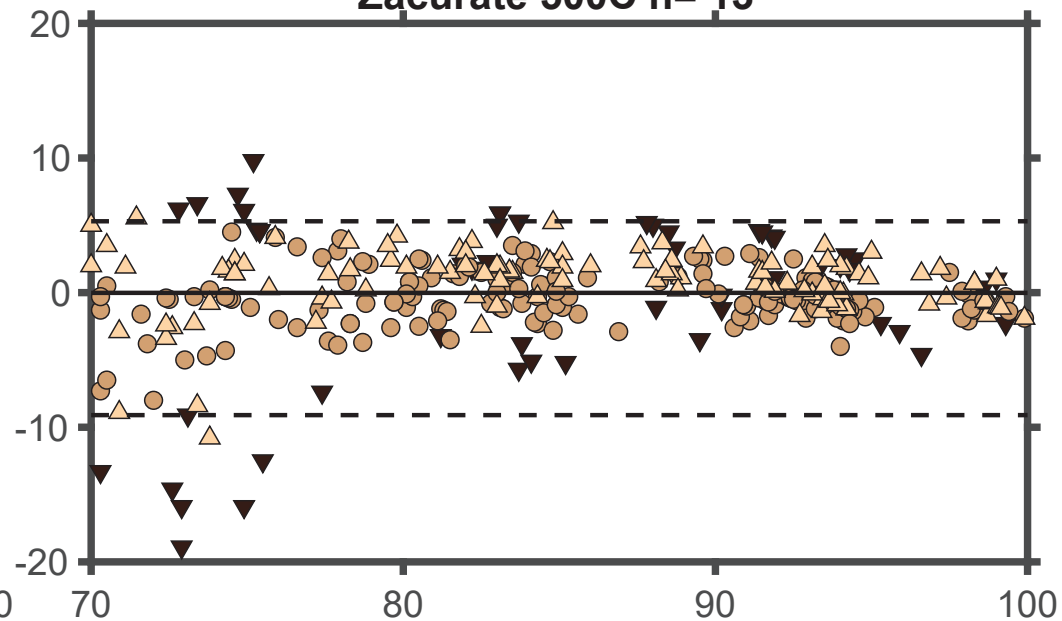

**Bodumed BDMOXMTRBLK n= 10**

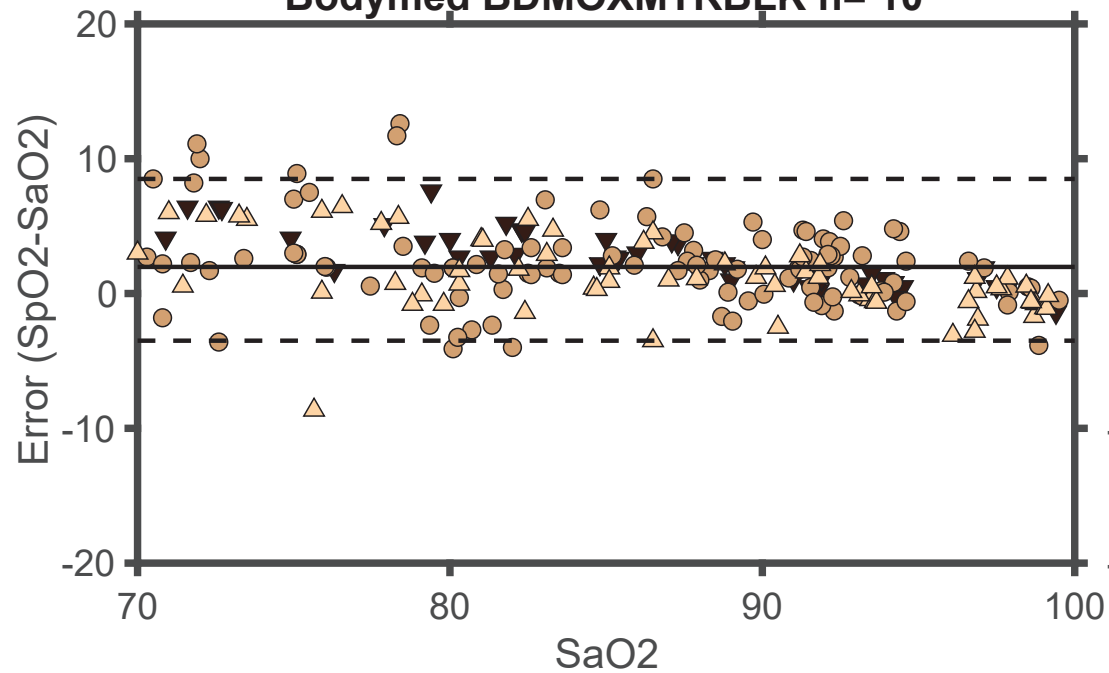

**CONTEC CMS50M n= 11**

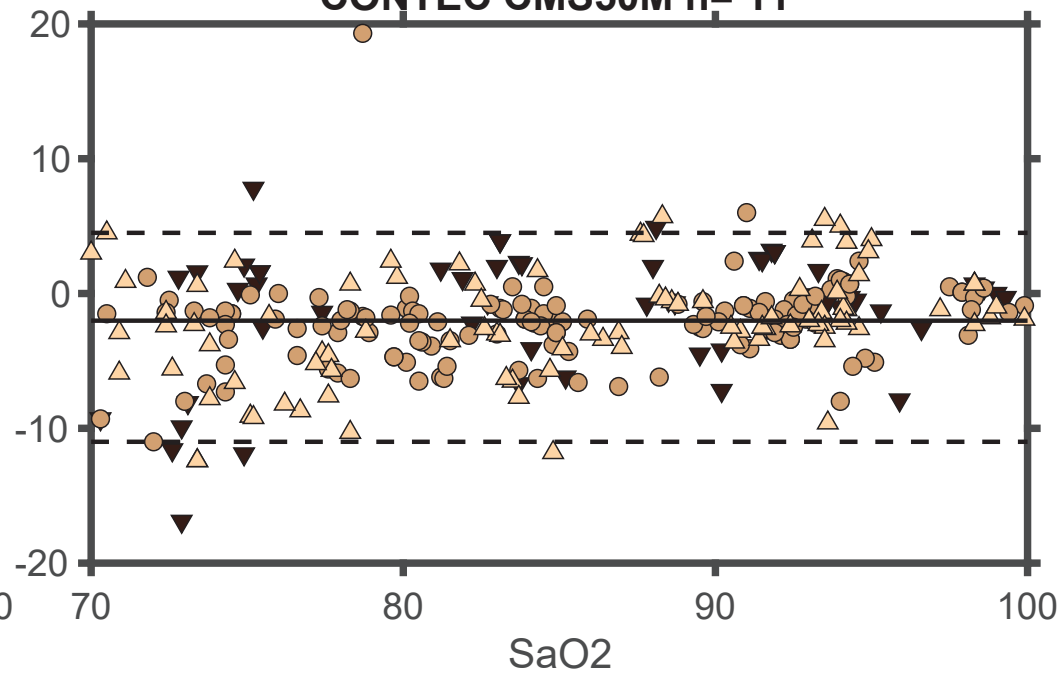

Supplement: Supplemental Figure S2 — Bland-Altman plot of four devices showing error vs SaO2. Error (SpO2 - SaO2) vs SaO2 for four of the POX. Error (pulse oximeter oxygen saturation [SpO2] − arterial blood oxygen saturation [SaO2]) is plotted against SaO2 measured by an ABL90 hemoximeter (Radiometer). For each device, data points are grouped by pigmentation (lightest third, medium third, and darkest third) as determined by ITA measured at the dorsal distal phalanx. Dashed horizontal lines are the upper and lower nonparametric limits of agreement, and horizontal solid line is the bias (mean of the error). [file mmc2.pdf]

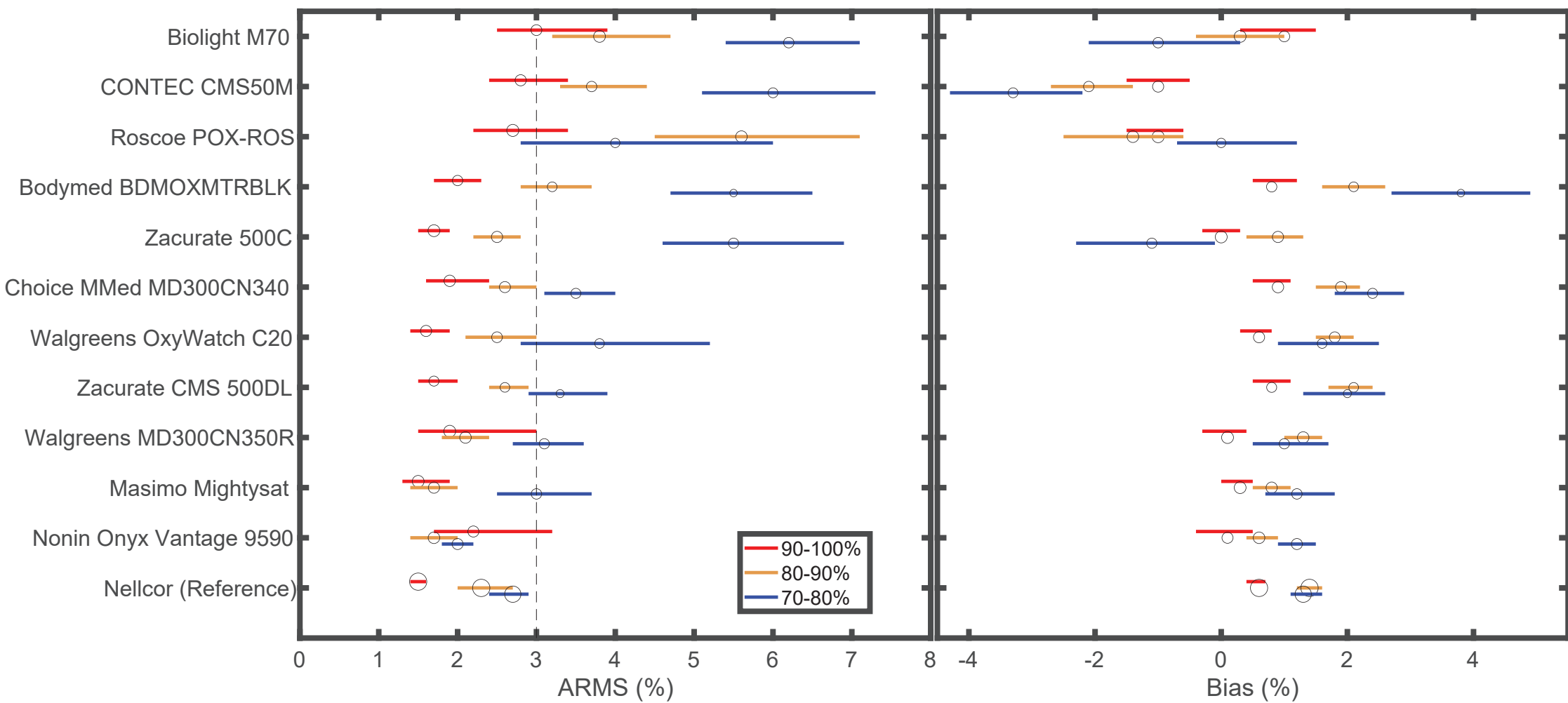

Supplement: Supplemental Figure S3 — Forest plot of ARMS and bias across difference SaO2 ranges. Forest plot showing ARMS and Bias for each device (lines show 95% confidence interval). Within each device the ARMS and bias are determined among ranges of SaO2 values: 70-80%, 80-90%, 90-100%. The dashed vertical line represents an ARMS of 3%. The size of the circles within each line is proportional to the number of measurements. [file mmc3.pdf]

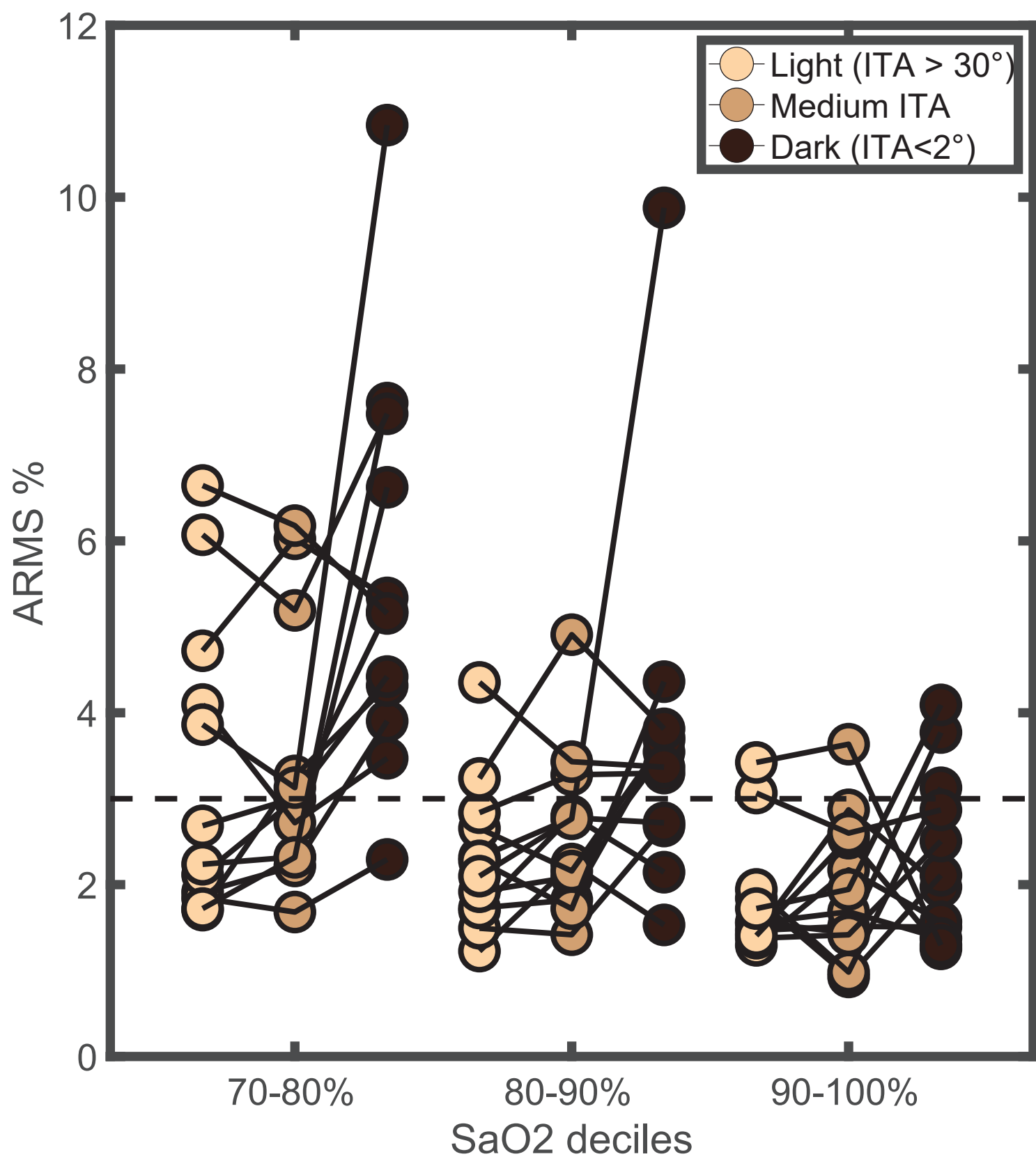

Supplement: Supplemental Figure S4 — Pulse oximeter performance (ARMS) by SaO2 decile and individual typology angle pigmentation ranges. For each device tested, subject data are grouped by SaO2 decile and ITA pigmentation ranges including lightest pigmented third of subjects (ITA >30°), medium pigmented third of subjects (30°>ITA>2°), and darkest pigmented third (ITA<2°), and ARMS is calculated for each device per decile and ITA pigmentation range. Each circle is the ARMS for a single device at a given SaO2 decile and ITA pigmentation range. Circles connected by lines represent ARMS values from the same device at a given SaO2 decile with varying ITA pigmentation range. [file mmc4.pdf]

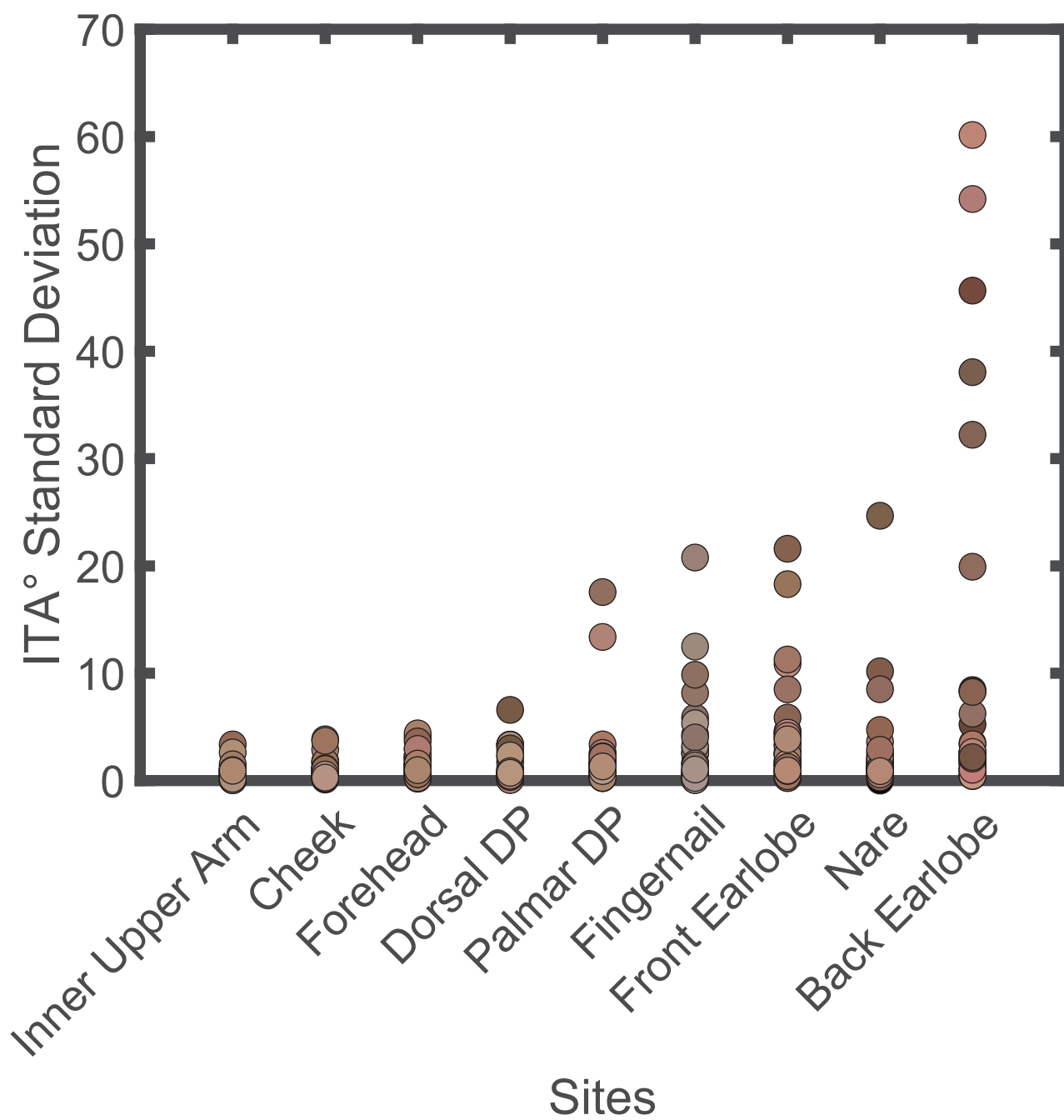

Supplement: Supplemental Figure S5 — Standard deviation of individual typology angle values captured across anatomical sites. Standard deviation between the three repeated ITA measurements shown by site. Colour used to represent data points is intended to represent study subject skin colour based on LAB values when transformed to RGB. The colour of each data point represents study subject skin colour measured by colorimetry but may not accurately portray perceived colour by a human observer. [file mmc5.pdf]

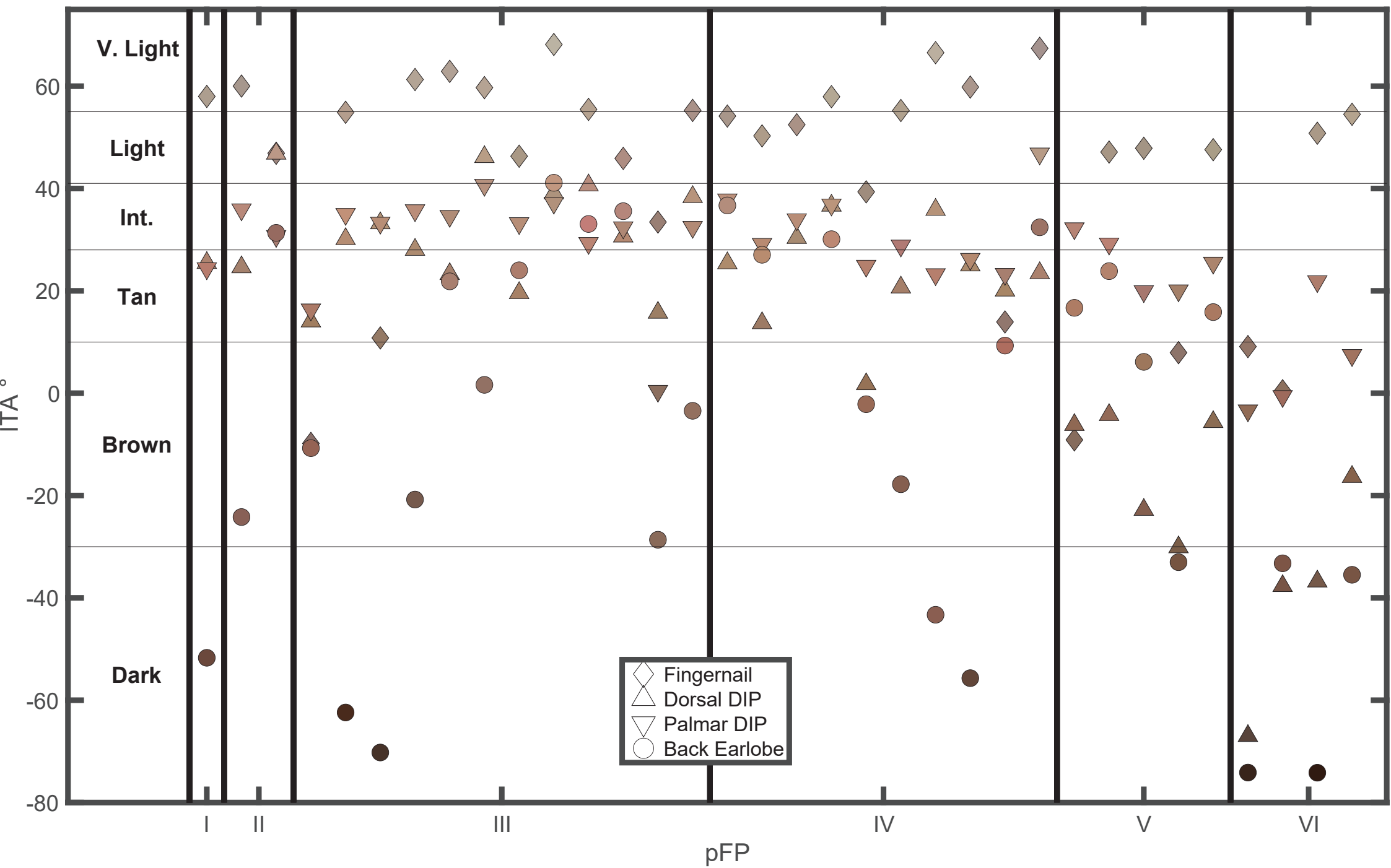

Supplement: Supplemental Figure S6 — Perceived Fitzpatrick Scale compared with individual typology angle measured at four anatomical sites. Inter- and intra-subject range of ITA values compared to pFP. ITA measured at four anatomical sites (fingernail, dorsal distal phalanx, palmar distal phalanx, back earlobe) compared to pFP for all subjects. Each vertical column of data points represents one subject. Colour used to represent data points is intended to represent study subject skin colour based on LAB values when transformed to RGB. Horizontal lines represent ITA cutoffs published by Del Bino et al. 2013 for skin phenotype (very light > 55° > light > 41° > intermediate > 28° > tan >10° > brown > −30° > dark) 25. Reproduced print and online figures may not portray accurate colour as intended. [file mmc6.pdf]

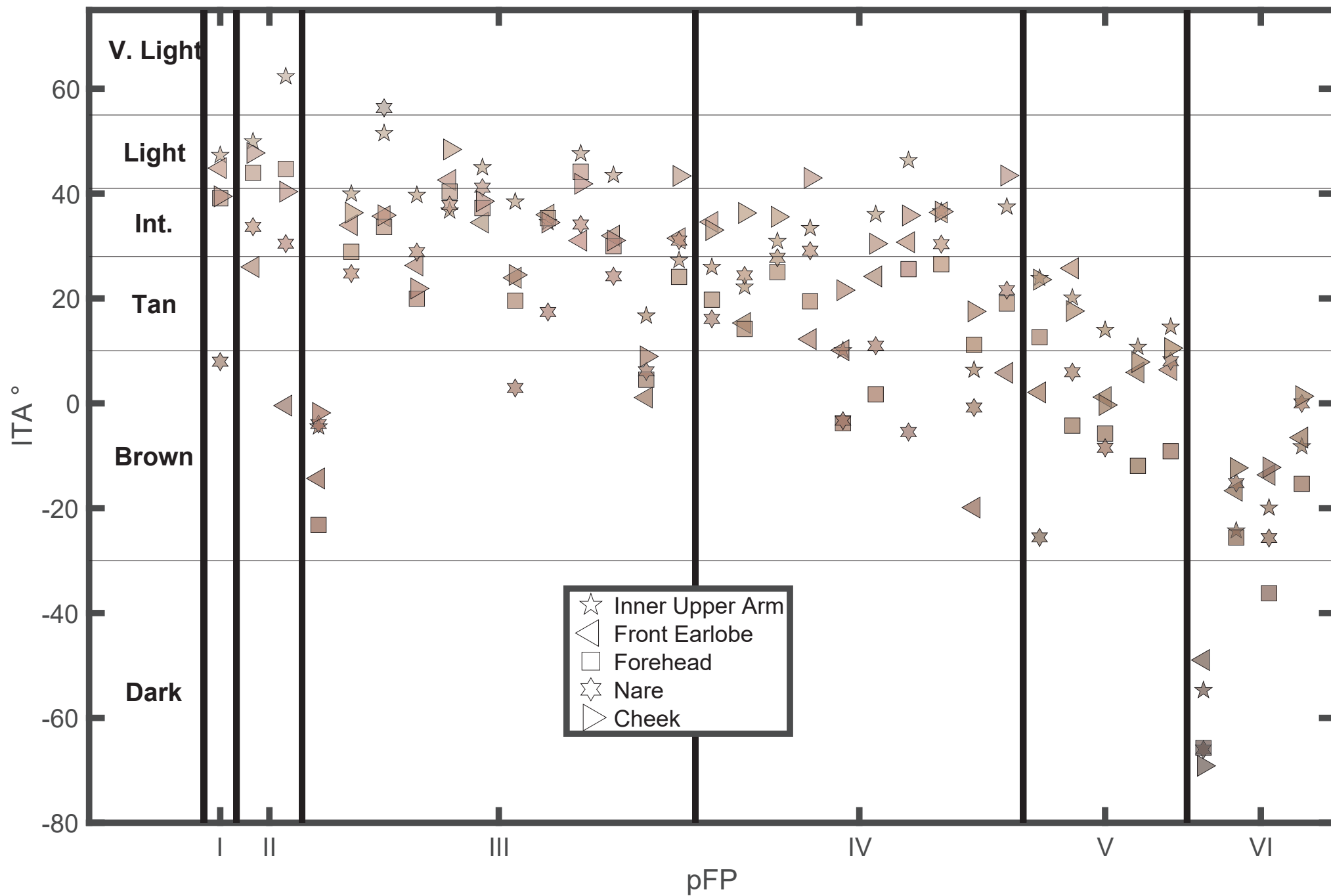

Supplement: Supplemental Figure S7 — Perceived Fitzpatrick Scale compared with individual typology angle measured at five anatomical sites. Inter- and intra-subject range of ITA values compared to pFP. ITA measured at five anatomical sites (inner upper arm, front earlobe, forehead, nare, cheek) compared to pFP for all subjects. Each vertical column of data points represents one subject. Colour used to represent data points is intended to represent study subject skin colour based on LAB values when transformed to RGB. Horizontal lines represent ITA cutoffs published by Del Bino et al. 2013 for skin phenotype (very light > 55° > light > 41° > intermediate > 28° > tan >10° > brown > −30° > dark)25. The colour of each data point represents study subject skin colour measured by colorimetry but may not accurately portray perceived colour by a human observer. [file mmc7.pdf]
