# Supplementary material for: The performance of 11 fingertip pulse oximeters during hypoxemia in healthy human participants with varied, quantified skin pigment
Source: eBioMedicine. 2024 Mar 8;102:105051. doi: 10.1016/j.ebiom.2024.105051 (PMC10943300; doi:10.1016/j.ebiom.2024.105051)
Supplement: Supplemental Figure S8 — Visualisation of forehead skin colour of study subjects. Forehead skin colour of study subjects by ITA and calculated RGB for each device tested. Skin colour represented in this figure is based on LAB values that are derived by the reflectance curves measured by the spectrophotometer, and the LAB values were transformed to RGB. The colour of each data point represents study subject skin colour measured by colorimetry but may not accurately portray perceived colour by a human observer. [file mmc8.pdf]

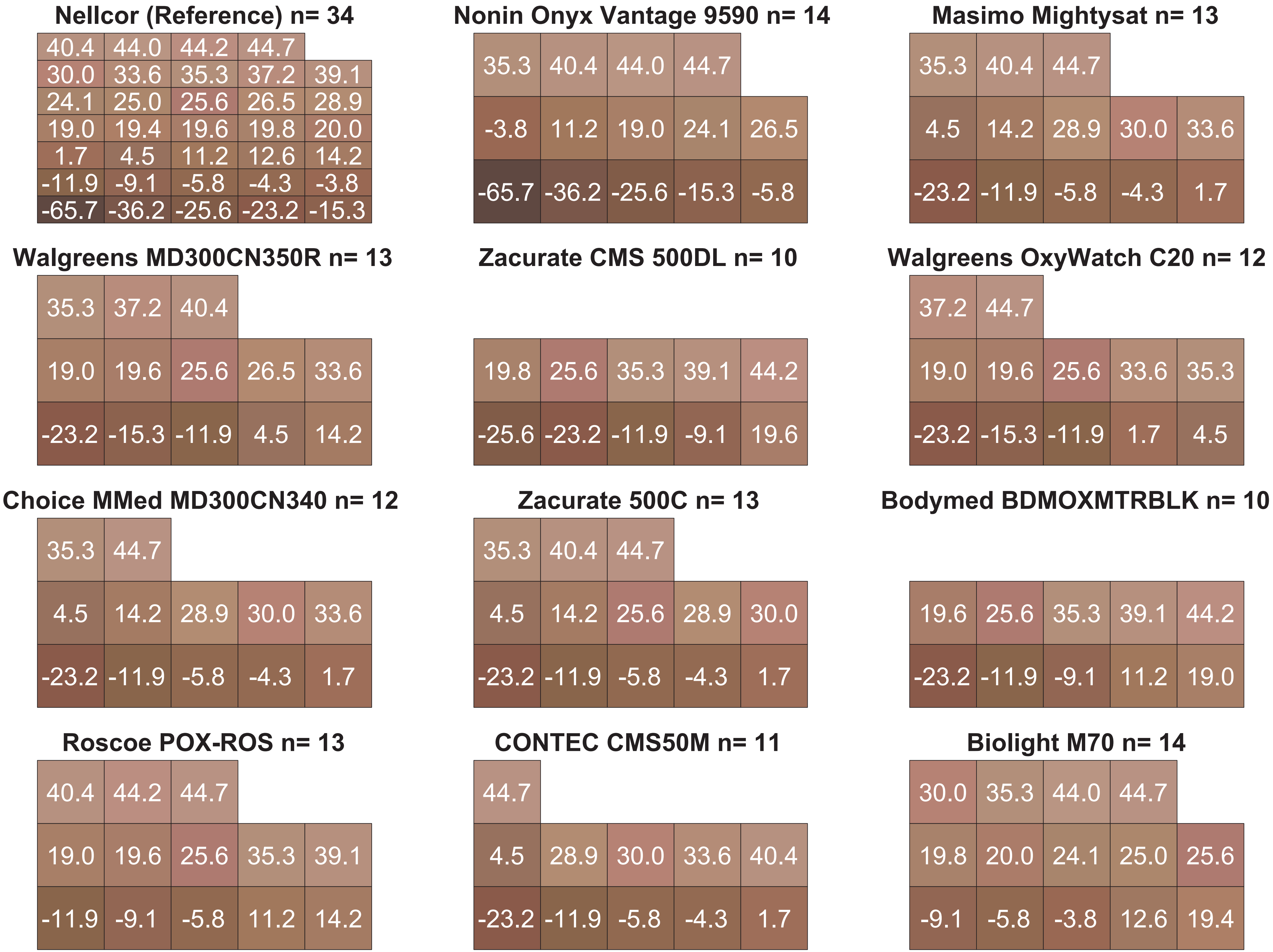
Nellcor (Reference) n= 34
40.4 44.0 44.2 44.7
30.0 33.6 35.3 37.2 39.1
24.1 25.0 25.6 26.5 28.9
19.0 19.4 19.6 19.8 20.0
1.7 4.5 11.2 12.6 14.2
-11.9 -9.1 -5.8 -4.3 -3.8
-65.7 -36.2 -25.6 -23.2 -15.3
Nonin Onyx Vantage 9590 n= 14
35.3 40.4 44.0 44.7
-3.8 11.2 19.0 24.1 26.5
-65.7 -36.2 -25.6 -15.3 -5.8
Masimo Mightysat n= 13
35.3 40.4 44.7
4.5 14.2 28.9 30.0 33.6
-23.2 -11.9 -5.8 -4.3 1.7
Walgreens MD300CN350R n= 13
35.3 37.2 40.4
19.0 19.6 25.6 26.5 33.6
-23.2 -15.3 -11.9 4.5 14.2
Zacurate CMS 500DL n= 10
19.8 25.6 35.3 39.1 44.2
-25.6 -23.2 -11.9 -9.1 19.6
Walgreens OxyWatch C20 n= 12
37.2 44.7
19.0 19.6 25.6 33.6 35.3
-23.2 -15.3 -11.9 1.7 4.5
Choice MMed MD300CN340 n= 12
35.3 44.7
4.5 14.2 28.9 30.0 33.6
-23.2 -11.9 -5.8 -4.3 1.7
Zacurate 500C n= 13
35.3 40.4 44.7
4.5 14.2 25.6 28.9 30.0
-23.2 -11.9 -5.8 -4.3 1.7
Bodymed BDMOXMTRBLK n= 10
19.6 25.6 35.3 39.1 44.2
-23.2 -11.9 -9.1 11.2 19.0
Roscoe POX-ROS n= 13
40.4 44.2 44.7
19.0 19.6 25.6 35.3 39.1
-11.9 -9.1 -5.8 11.2 14.2
CONTEC CMS50M n= 11
44.7
4.5 28.9 30.0 33.6 40.4
-23.2 -11.9 -5.8 -4.3 1.7
Biolight M70 n= 14
30.0 35.3 44.0 44.7
19.8 20.0 24.1 25.0 25.6
-9.1 -5.8 -3.8 12.6 19.4
