# Supplementary material for: The performance of 11 fingertip pulse oximeters during hypoxemia in healthy human participants with varied, quantified skin pigment
Source: eBioMedicine. 2024 Mar 8;102:105051. doi: 10.1016/j.ebiom.2024.105051 (PMC10943300; doi:10.1016/j.ebiom.2024.105051)
Supplement: Supplemental Table S1 — ITA range for each pFP. [file mmc9.docx]

| pFP | Number of Subjects | Site | ITA° Range | ITA° median (IQR Q1, Q3) |
| --- | --- | --- | --- | --- |
| I | 1 | Fingernail | 58.0 | 58.0 |
|  |  | Dorsal DIP | 25.5 | 25.5 |
|  |  | Palmar DIP | 24.4 | 24.4 |
|  |  | Inner Upper Arm | 47.3 | 47.3 |
|  |  | Front Earlobe | 44.9 | 44.9 |
|  |  | Back Earlobe | -51.7 | -51.7 |
|  |  | Forehead | 39.1 | 39.1 |
|  |  | Nare | 7.9 | 7.9 |
|  |  | Cheek | 39.5 | 39.5 |
| II | 2 | Fingernail | 46.9, 60.0 | 53.5 (46.9, 60.0) |
|  |  | Dorsal DIP | 24.6, 46.8 | 35.7 (24.6, 46.8) |
|  |  | Palmar DIP | 30.7, 35.9 | 33.3 (30.7, 35.9) |
|  |  | Inner Upper Arm | 49.9, 62.3 | 56.1 (49.9, 62.3) |
|  |  | Front Earlobe | -0.4, 26.0 | 12.8 (-0.4, 26.0) |
|  |  | Back Earlobe | -24.2, 31.3 | 3.6 (-24.2, 31.3) |
|  |  | Forehead | 44.0, 44.7 | 44.3 (44.0, 44.7) |
|  |  | Nare | 30.5, 33.7 | 32.1 (30.5, 33.7) |
|  |  | Cheek | 40.4, 47.8 | 44.1 (40.4, 47.8) |
| III | 12 | Fingernail | -9.8, 68.1 | 55.1 (39.7, 60.5) |
|  |  | Dorsal DIP | 14.0, 46.2 | 30.4 (21.4, 38.7) |
|  |  | Palmar DIP | 0.4, 40.7 | 33.2 (30.9, 35.4) |
|  |  | Inner Upper Arm | -4.5, 51.5 | 39.1 (30.9, 44.3) |
|  |  | Front Earlobe | -14.3, 42.6 | 31.7 (25.1, 35.1) |
|  |  | Back Earlobe | -70.2, 41.1 | -0.9 (-24.7, 28.5) |
|  |  | Forehead | -23.2, 44.2 | 29.4 (19.8, 36.3) |
|  |  | Nare | -3.9, 56.3 | 26.8 (11.8, 36.0) |
|  |  | Cheek | -1.8, 48.4 | 35.2 (23.2, 40.2) |
| IV | 10 | Fingernail | 13.9, 67.4 | 54.7 (50.3, 59.8) |
|  |  | Dorsal DIP | 1.8, 36.7 | 24.2 (20.1, 30.4) |
|  |  | Palmar DIP | 23.2, 46.8 | 29.0 (24.9, 36.8) |
|  |  | Inner Upper Arm | 6.4, 46.3 | 32.2 (22.2, 36.6) |
|  |  | Front Earlobe | -82.0, 36.4 | 13.8 (5.8, 30.7) |
|  |  | Back Earlobe | -83.2, 36.7 | 3.6 (-43.3, 30.1) |
|  |  | Forehead | -3.8, 26.5 | 19.2 (11.2, 25.0) |
|  |  | Nare | -5.5, 30.4 | 18.9 (-0.8, 27.8) |
|  |  | Cheek | 17.5, 43.5 | 35.7 (30.4, 36.6) |
| V | 5 | Fingernail | -9.1, 47.9 | 47.1 (3.7, 47.7) |
|  |  | Dorsal DIP | -30.1, -4.2 | -6.2 (-24.6, -5.3) |
|  |  | Palmar DIP | 19.9, 32.2 | 25.5 (20.0, 30.0) |
|  |  | Inner Upper Arm | 10.7, 23.9 | 14.6 (13.2, 21.1) |
|  |  | Front Earlobe | 1.2, 25.8 | 5.9 (1.9, 11.2) |
|  |  | Back Earlobe | -33.0, 23.9 | 15.9 (-3.7, 18.5) |
|  |  | Forehead | -11.9, 12.6 | -5.8 (-9.8, -0.0) |
|  |  | Nare | -89.4, 8.0 | -8.5 (-41.5, 6.5) |
|  |  | Cheek | -0.3, 23.6 | 10.5 (5.8, 19.1) |
| VI | 4 | Fingernail | 0.5, 54.5 | 30.0 (4.8, 52.7) |
|  |  | Dorsal DIP | -67.0, -16.3 | -37.2 (-52.3, -26.6) |
|  |  | Palmar DIP | -3.4, 21.9 | 3.4 (-2.0, 14.6) |
|  |  | Inner Upper Arm | -54.7, -8.2 | -22.1 (-39.5, -14.1) |
|  |  | Front Earlobe | -49.0, -6.5 | -15.2 (-32.8, -10.1) |
|  |  | Back Earlobe | -74.2, -33.2 | -54.8 (-74.2, -34.4) |
|  |  | Forehead | -65.7, -15.3 | -30.9 (-51.0, -20.5) |
|  |  | Nare | -66.0, -0.0 | -20.4 (-45.9, -7.6) |
|  |  | Cheek | -69.1, 1.4 | -12.3 (-40.7, -5.4) |
